# What makes a histone variant a variant: Changing H2A to become H2A.Z

**DOI:** 10.1371/journal.pgen.1009950

**Published:** 2021-12-06

**Authors:** Hilary T. Brewis, Alice Y. Wang, Aline Gaub, Justine J. Lau, Peter C. Stirling, Michael S. Kobor

**Affiliations:** 1 Centre for Molecular Medicine and Therapeutics, BC Children’s Hospital Research Institute, Department of Medical Genetics, University of British Columbia, Vancouver, Canada; 2 Terry Fox Laboratory, BC Cancer, Department of Medical Genetics, University of British Columbia, Vancouver, Canada; Institute of Functional Epigenetics, GERMANY

## Abstract

Chromatin structure and underlying DNA accessibility is modulated by the incorporation of histone variants. H2A.Z, a variant of the H2A core histone family, plays a distinct and essential role in a diverse set of biological functions including gene regulation and maintenance of heterochromatin-euchromatin boundaries. Although it is currently unclear how the replacement of H2A with H2A.Z can regulate gene expression, the variance in their amino acid sequence likely contributes to their functional differences. To tease apart regions of H2A.Z that confer its unique identity, a set of plasmids expressing H2A-H2A.Z hybrids from the native H2A.Z promoter were examined for their ability to recapitulate H2A.Z function. First, we found that the H2A.Z M6 region was necessary and sufficient for interaction with the SWR1-C chromatin remodeler. Remarkably, the combination of only 9 amino acid changes, the H2A.Z M6 region, K79 and L81 (two amino acids in the α2-helix), were sufficient to fully rescue growth phenotypes of the *htz1Δ* mutant. Furthermore, combining three unique H2A.Z regions (K79 and L81, M6, C-terminal tail) was sufficient for expression of H2A.Z-dependent heterochromatin-proximal genes and *GAL1* derepression. Surprisingly, hybrid constructs that restored the transcription of H2A.Z-dependent genes, did not fully recapitulate patterns of H2A.Z-specific enrichment at the tested loci. This suggested that H2A.Z function in transcription regulation may be at least partially independent of its specific localization in chromatin. Together, this work has identified three regions that can confer specific H2A.Z-identity to replicative H2A, furthering our understanding of what makes a histone variant a variant.

## Author summary

Chromatin structure in eukaryotes is integral to all aspects of genome function. Many essential biological processes depend on chromatin remodeling for regulating access to DNA such as transcription, replication, and DNA repair. One highly conserved method of chromatin remodeling is the replacement of the replicative histones with a histone variant. Unlike replicative histones, which are distributed equally in chromatin during DNA replication, histone variants can be transcribed and deposited throughout the cell cycle at specific locations. Once incorporated, small differences in a histone variant’s protein sequence and structure can result in unique chromatin neighbourhoods with distinct character and function from the replicative histone. Therefore, to better understand the role of a histone variant, it is important to define how its protein sequence contributes to its unique identity. Here, using a hybrid gene approach in budding yeast, we systematically determine which amino acids contribute to H2A.Z unique identity as a histone variant.

## Introduction

In eukaryotic cells, chromatin structure is a major modulator of genomic processes that require DNA accessibility. The basic packaging unit of chromatin is the nucleosome, consisting of approximately 146 base pairs of DNA wrapped around a protein octamer composed of two copies of each of the core histones: H2A, H2B, H3, and H4 [1]. One modulator of chromatin structure is the incorporation of histone variants [2].Unlike the core histones (also called replicative histones), which are produced in equal amounts during S-phase by multiple genes, histone variants are encoded by one or two replication-independent genes, allowing variant-specific transcription and deposition throughout the cell cycle [3]. Histone variants are also distinct in their protein sequence and structure, which can lead to different post-translational modifications, recruitment of proteins to chromatin that specifically interact with the variant, and changes to the stability and mobility of the nucleosome into which they are incorporated [4]. Through these properties, histone variants are capable of establishing unique chromatin neighbourhoods with distinct character and function in a regulated manner.

H2A.Z is a variant of the H2A histone family present in almost all eukaryotic organisms, with 65% sequence conservation between *Saccharomyces cerevisiae* and humans [5]. Unlike replicative H2A, which diverged multiple times in different lineages, H2A.Z had a single evolutionary origin and has remained distinct from replicative H2A ever since [6]. Essential in mammalian cells, H2A.Z has crucial roles in the regulation of gene expression, DNA repair, chromosome segregation, and maintenance of heterochromatin-euchromatin boundaries [5,7,8]. As a result, H2A.Z has been associated with a diverse set of biological functions in mammals [9] including memory [10–14], the epithelial-to-mesenchymal transition [15,16], embryonic development [17], and cell proliferation in cancer [18–21].

In *S*. *cerevisiae*, H2A.Z is incorporated into chromatin by the SWR1 complex (SWR1-C), a highly conserved ATP-dependent chromatin-remodeler [22–25], with the help of two histone chaperones, Nap1 and Chz1 [26–30]. Intriguingly, the growth defects of H2A.Z knockouts are alleviated by the simultaneous deletion of SWR1-C subunits, suggesting SWR1-C has erroneous activity when missing its H2A.Z substrate (apo-SWR1-C) [31–33]. H2A.Z replaces replicative H2A in 5–10% of nucleosomes and is enriched near centromeres, at the border of heterochromatic domains, and primarily at the +1 nucleosome at the transcription start site at approximately 63% of gene promoters under basal conditions [34–39]. While there is substantial evidence that H2A.Z occupancy influences gene expression, the exact mechanism of its involvement in transcription regulation remains an active area of research [7]. One potential model suggests that H2A.Z is involved in gene poising and activation [4,7]. Various studies in *Drosophila*, plants, and mammalian tissues, have found that H2A.Z occupies the promoter in the absence of gene expression, but decreases in enrichment upon gene induction [40–45]. Furthermore, cells lacking H2A.Z have defective activation of the inducible genes *GAL1*, *PHO5*, and *PUR5* in *S*. *cerevisiae* [31,46–49].

H2A.Z’s primary protein sequence is remarkably similar to H2A’s, with 60% identical amino acids between the two proteins, including in some of the core structural features [50]. Despite this similarity, H2A.Z is encoded by a single non-essential gene in *S*. *cerevisiae* (*HTZ1*) whereas the deletion of both H2A genes (*HTA1* and *HTA2*) is lethal [51,52]. However, the *htz1Δ* mutant has various growth phenotypes, including drug sensitivities that cannot be rescued by overexpression of H2A or by H2A expressed from an H2A.Z promoter [51,53]. This suggests that amino acid composition, not expression dynamics, is the main driver of H2A.Z’s unique function from H2A. The overall structure of a H2A.Z-containing nucleosome is very similar to that of a nucleosome containing H2A, which is unsurprising given the similarities between the two histone’s 3D conformations [50,54]. Regardless, *in vitro*, H2A.Z nucleosomes have a lower physical breaking force and thermal stability than H2A nucleosomes [54–56], and salt washes indicate that H2A.Z is more readily lost from chromatin than H2A [37,54,57,58]. Therefore, while these histones have similar structures, the regions of primary sequence divergence between H2A and H2A.Z likely do give rise to their unique functions.

Distinguishing which of H2A.Z’s unique regions are responsible for its identity is key to understanding its functional specialization and may provide new insights into how the replacement of replicative H2A with H2A.Z can regulate gene expression and overall chromatin biology. Previous studies have explored the function of H2A.Z’s primary structure using a variety of methods including gene truncations, alanine point mutation libraries, and genetic screens of H2A.Z mutants that are not incorporated into chromatin [53,59,60]. However, while these methods are effective at identifying amino acids required for H2A.Z function, they do not determine which ones are responsible for H2A.Z’s unique identity. To determine which regions distinguish H2A.Z from H2A, we built a plasmid library consisting of various H2A/H2A.Z hybrid constructs created by replacing regions of the H2A gene (*HTA1*) with the corresponding H2A.Z gene (*HTZ1*) and *vice versa*. We identified three regions that were able to confer H2A.Z identity: the M6 region, the C-terminal tail, and two evolutionarily conserved amino acids in the α2 helix (K79 and L81). Remarkably, the combination of only 9 amino acid changes, the H2A.Z M6 region, K79 and L81, was sufficient to fully rescue *htz1Δ* growth phenotypes. However, the addition of the H2A.Z C-terminal tail enabled wild-type transcription of H2A.Z-dependent heterochromatin-proximal genes and *GAL1* after derepression. Surprisingly, hybrid constructs that restored the mRNA levels of H2A.Z-dependent genes had chromatin enrichment patterns that more closely resembled H2A than H2A.Z, suggesting that H2A.Z function in transcription regulation may be at least partially independent of its specific pattern of localization in the genome. More generally, we took advantage of the functional differences between H2A and H2A.Z, as well as the power of yeast genetics, to broadly explore what makes a histone variant a variant.

## Results

### H2A/H2A.Z hybrid constructs revealed regions that contribute to H2A.Z identity

**Fig 1:**
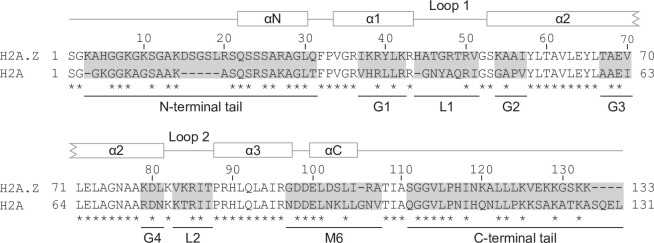
Amio acids that differ between H2A.Z and H2A were grouped into nine distinct regions. Sequence alignment of the *S*. *cerevisiae* H2A.Z and H2A proteins with conserved amino acids indicated by asterisks. Regions that differ between H2A.Z and H2A were divided into nine distinct regions: N-terminal tail, C-terminal tail, M6, two histone loops (L1 and L2) and four groups in the histone fold (G1-G4). The H2A.Z secondary structure is shown above the sequence alignment with lines indicating unstructured flexible regions and boxes indicating alpha helices.

**Fig 2:**
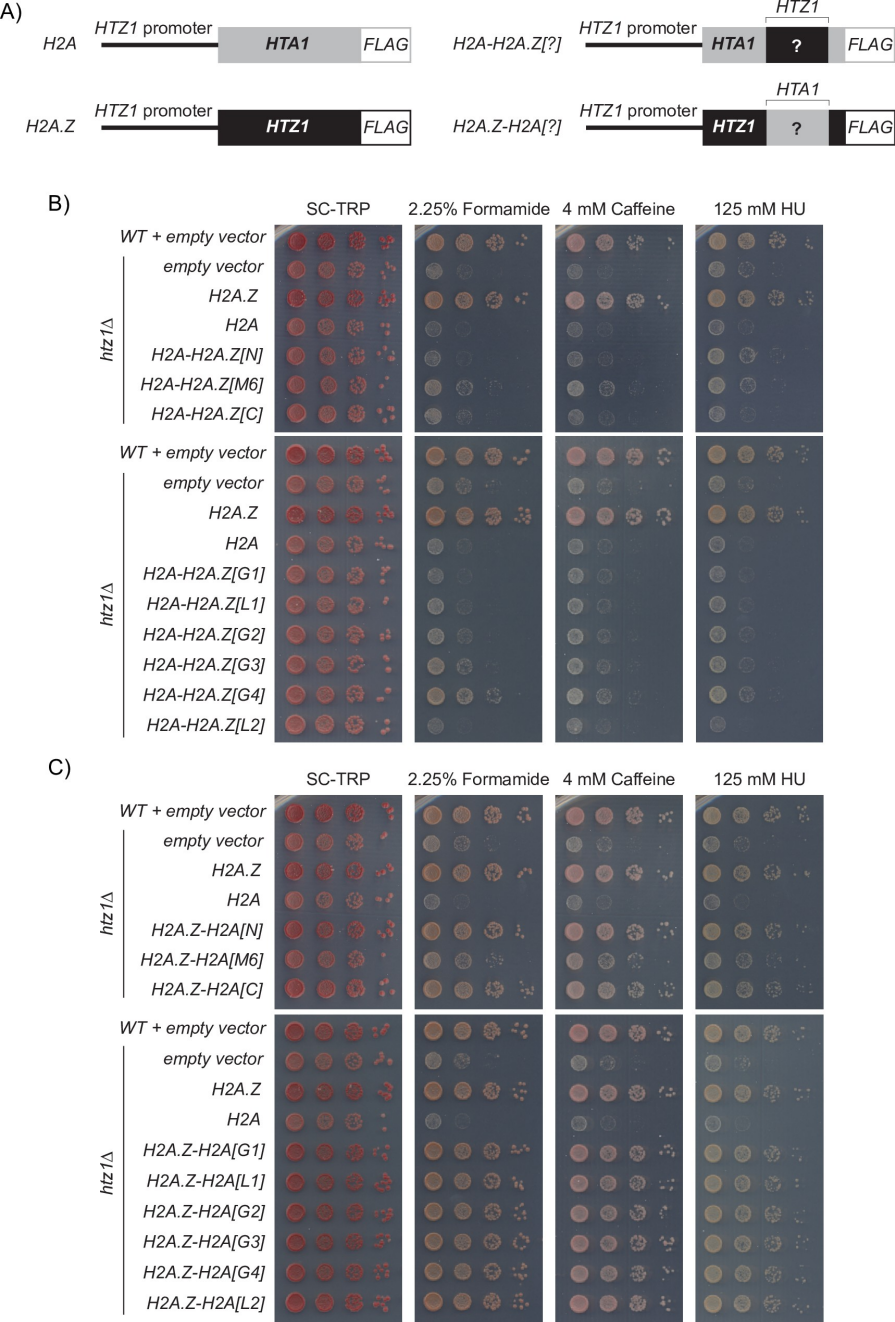
Systematic analysis of regions that diverge between H2A and H2A.Z revealed that the N-terminal, M6, G3, and G4 regions of H2A.Z may contribute to H2A.Z-specific function. (A) H2A.Z (*HTZ1*, black) and H2A (*HTA1*, grey) parent constructs were used to create the hybrid H2A.Z-H2A and H2A-H2A.Z constructs. Each construct was expressed from the native H2A.Z gene promoter and C-terminally *3xFLAG* tagged. (B) Growth assays of the *H2A-H2A*.*Z* mutants indicated that mutants containing either the H2A.Z N-terminus, M6, G3, or G4 region had improved growth over the *H2A* mutant, while (C) the *H2A*.*Z-H2A* mutants showed that of the nine regions of H2A.Z, only the M6 region could not be functionally replaced by the corresponding region from H2A. Cells expressing the indicated hybrid constructs were 10-fold serially diluted, spotted onto SC-TRP media with the indicated concentrations of formamide, caffeine, and hydroxyurea and grown for 3 days.

In order to determine which protein regions are involved in conferring H2A.Z-specific functions, we grouped all of the amino acids that differ between H2A.Z and H2A into nine regions (Fig 1). One of these regions, M6, is a group of 11 amino acids immediately preceding the C-terminal tail that has previously been shown to be a defining feature of H2A.Z identity in *S*. *cerevisiae* and *Drosophila* [61,62]. Apart from the M6 region, the boundaries of each of the regions in this study were determined by H2A.Z’s primary and secondary structure. We constructed hybrid constructs using two previously characterized parent plasmids, *H2A*.*Z* and *H2A*, which are both expressed from the *H2A*.*Z* gene promoter (*HTZ1*) and C-terminally FLAG-tagged for determination of protein levels and to enable functional genomic studies (Fig 2A) [53]. To determine if a region was sufficient to confer H2A.Z identity onto H2A, we built a hybrid construct of H2A containing the corresponding region of H2A.Z (H2A-H2A.Z), while a hybrid construct of H2A.Z with the equivalent H2A region (H2A.Z-H2A) was used to determine if a region was necessary for H2A.Z function. In order to assess the ability of each region to recapitulate several aspects of H2A.Z identity, these hybrid constructs were placed into an *htz1Δ* background. While the *H2A*.*Z* mutant showed a marginal increase in H2A.Z abundance in comparison to wild-type (S1A Fig), all hybrid constructs were present in comparable levels to the H2A.Z construct (S1B and S1C Fig).

Given that the *htz1Δ* mutant exhibits slow growth when exposed to a variety of genotoxic stress conditions [23,24], we used growth assays to assess how exchanging each of the nine H2A.Z and H2A regions impacted cell fitness. Consistent with previous reports [51,53], we found that the *htz1Δ* mutant had growth defects in formamide, caffeine, and hydroxyurea (HU), which could not be rescued by expressing H2A from the H2A.Z promoter (Figs 2B and S2A). In contrast, the *H2A*.*Z* mutant restored cell growth and was indistinguishable from the wild-type empty vector control. A visual comparison of the H2A-H2A.Z hybrid mutants growth phenotypes indicated that several regions could partially recapitulate H2A.Z identity. The *H2A-H2A*.*Z[N]*, *H2A-H2A*.*Z[M6]*, *H2A-H2A*.*Z[G3]*, *H2A-H2A*.*Z[G4]* mutants all showed improved growth over the *H2A* mutant in various conditions. However, no single region was able to fully rescue growth phenotypes of the *htz1Δ* mutant and thus fully recapitulate H2A.Z identity. Therefore, some combination of these nine unique regions must be required to completely confer H2A.Z function.

To determine which of the nine regions were necessary for H2A.Z’s identity, we compared the growth of each H2A.Z-H2A hybrid mutant to the *H2A*.*Z* mutant. The *H2A*.*Z-H2A[M6]* mutant had growth defects in all three conditions in comparison to the *H2A*.*Z* mutant (Figs 2C and S2B). All of the remaining H2A.Z-H2A hybrid mutants grew similar to wild-type. Therefore, of the nine divergent regions between H2A and H2A.Z, only the M6 region was necessary for H2A.Z dependent phenotypes.

### The H2A.Z M6 region was necessary and sufficient for interaction with SWR1-C and histone chaperones

**Fig 3:**
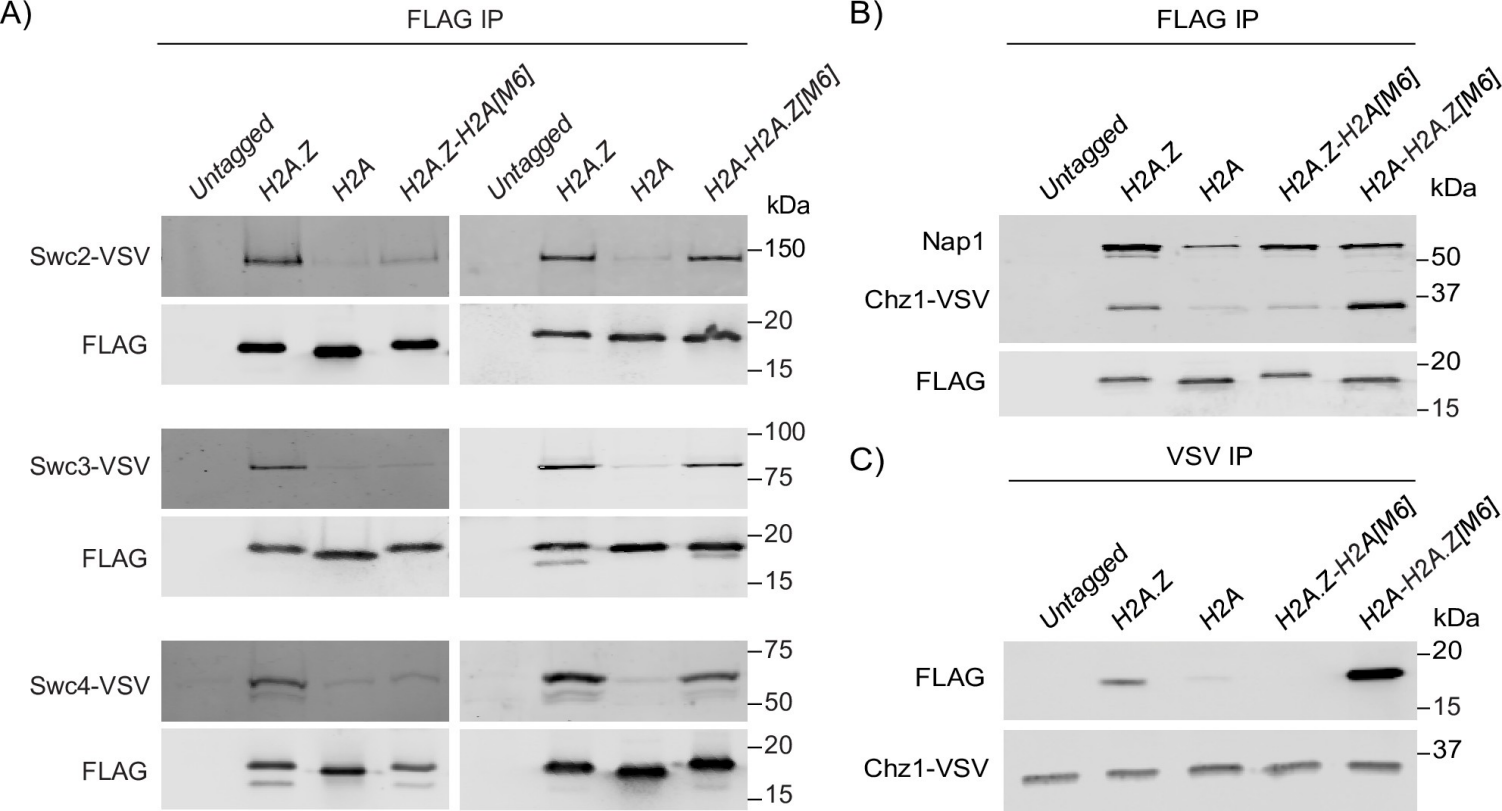
The H2A.Z M6 region was necessary and sufficient for interaction with known H2A.Z partners. (A) Immunoprecipitation of the FLAG-tagged hybrid proteins revealed that the M6 region of H2A.Z was necessary and sufficient for co-purification with VSV-tagged SWR1-C subunits. (B) Immunoprecipitation of the FLAG-tagged hybrid constructs revealed M6 as a key region for H2A.Z interaction with the histone chaperones Nap1 and VSV-tagged Chz1. (C) The reciprocal immunoprecipitation of VSV-tagged Chz1 confirmed an increase in interaction with the H2A-H2A.Z construct in comparison to the H2A.Z construct.

The H2A.Z M6 region is a key binding region for several factors involved in H2A.Z’s incorporation into chromatin, including SWR1-C and the histone chaperones Nap1 and Chz1 [27,61,63,64]. Therefore, the growth phenotypes observed for the *H2A*.*Z-H2A[M6]* and *H2A-H2A*.*Z[M6]* mutants may be associated with how the hybrid constructs interact with these proteins. To examine this possibility more directly, we purified the FLAG-tagged M6 hybrid constructs from cells containing VSV-tagged SWR1-C subunits: Swc2, Swc3, or Swc4. Consistent with previous work, all the tested subunits co-purified with the H2A and H2A.Z-H2A[M6] constructs at lower levels compared to the H2A.Z construct, confirming that the H2A.Z M6 region is required for interaction with SWR1-C (Figs 3A and S3A and S3B) [61,63,64]. In contrast, the H2A-H2A.Z[M6] construct associated with each SWR1-C subunit in a manner that was reminiscent of the H2A.Z construct. Therefore, as judged by this analytical method, the H2A.Z M6 region on its own was sufficient for interaction with SWR1-C subunits.

Purifications of the FLAG-tagged constructs in cells with VSV-tagged Chz1 showed that the H2A.Z-H2A[M6] construct closely resembled the H2A construct, which indicated that the H2A.Z M6 region was necessary for Chz1 interaction with H2A.Z (Figs 3B and S3C). The H2A-H2A.Z[M6] construct, however, had an increased interaction with Chz1 in comparison to the H2A.Z construct, a result which replicated in reciprocal purifications of VSV-tagged Chz1 (Figs 3C and S3D). This may suggest that H2A.Z amino acids outside the M6 region partially inhibited interaction with Chz1. Conversely, Nap1 interaction with H2A.Z was not entirely dependent on the H2A.Z M6 region (Figs 3B and S3C). While the H2A.Z-H2A[M6] construct did have reduced interaction with Nap1 in comparison to the H2A.Z construct, it also had increased interaction in comparison to the H2A construct. In addition, the H2A-H2A.Z[M6] construct did not fully recapitulate H2A.Z interaction with Nap1. Therefore, while the H2A.Z M6 region did play a role in interaction with Nap1, other H2A.Z regions may be required for complete wild-type Nap1 association.

### In combination with M6, an evolutionarily conserved region in the α2-helix of H2A.Z confers H2A.Z-specific growth phenotypes

**Fig 4:**
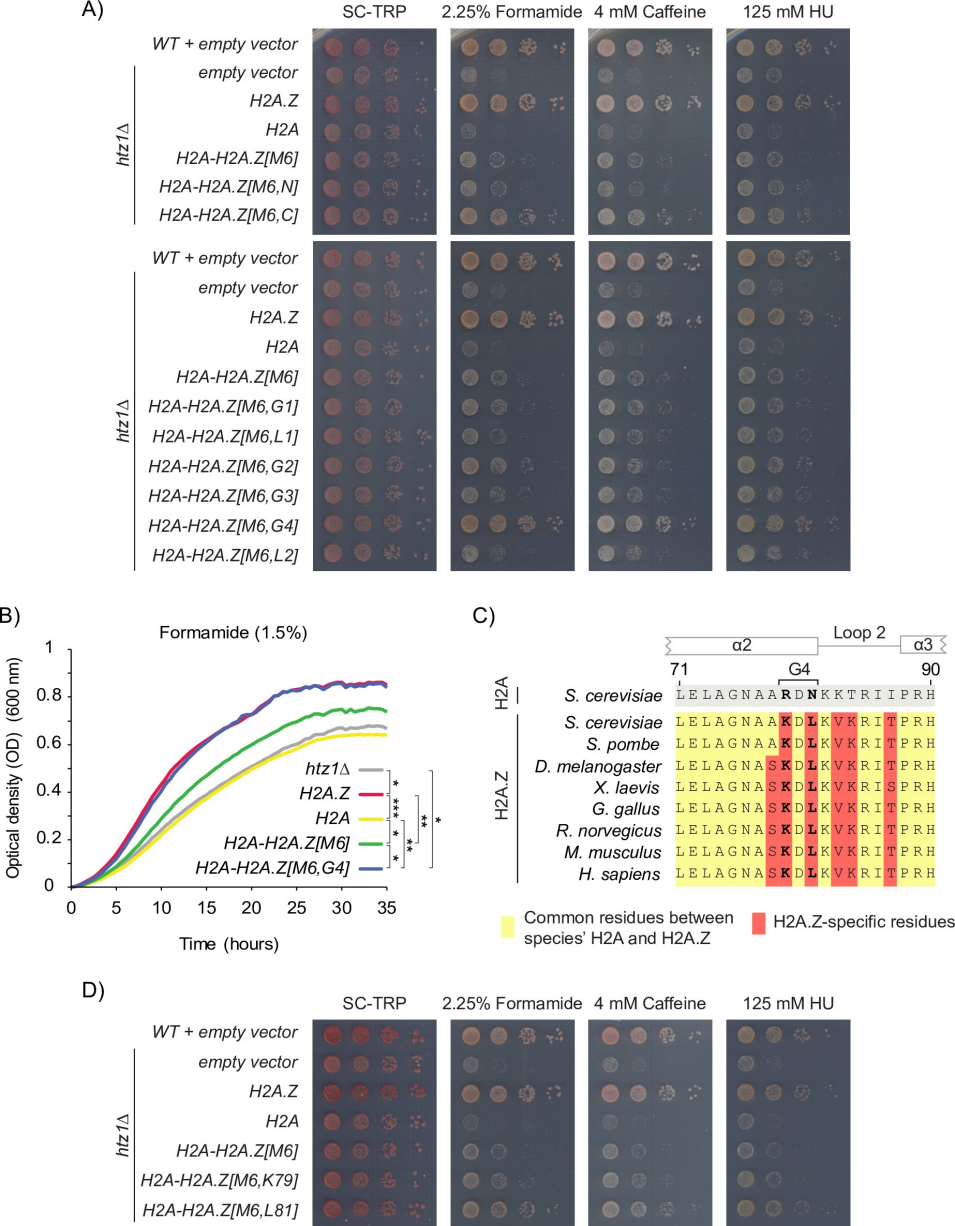
In combination with M6, an evolutionary conserved region in the alpha 2 helix of H2A.Z distinguished the histone variant H2A.Z from replicative H2A and conferred H2A.Z-specific growth phenotypes. (A) The *H2A-H2A*.*Z[M6*,*G4]* mutant had comparable growth to the *H2A*.*Z* mutant. Cells expressing the indicated hybrid constructs were 10-fold serially diluted, spotted onto SC-TRP media with the indicated concentrations of formamide, caffeine, and hydroxyurea and grown for 3 days. (B) The growth curves of the *H2A*.*Z* and *H2A-H2A*.*Z[M6*,*G4]* mutants were indistinguishable. Each curve was generated by taking the average OD (measured every 20 minutes) of three biological replicates grown in SC-TRP liquid media in the presence of 1.5% formamide. Asterisks indicate significant comparisons determined by comparing the area under the curve of for each mutant with unpaired one-tailed Student’s *t*-tests. * p-value <0.05, ** p-value <0.01, *** p-value <0.001. (C) Sequence alignment of the H2A.Z L2 loop region across species revealed that amino acids K79 and L81 (bold) are highly conserved among H2A.Z homologs and are consistently divergent from H2A. Amino acids in yellow indicate that it is found in both H2A.Z and H2A for the indicated species. Red indicates that the amino acid is unique to H2A.Z in the indicated species. (D) The growth phenotypes of the *H2A-H2A*.*Z[M6*,*G4]* mutant were primarily driven by L81. Growth assays were performed as previously described in panel “A”.

As our H2A-H2A.Z hybrid constructs showed that no single H2A.Z region conferred upon a H2A background was able to fully rescue the *htz1Δ* mutant growth defects and recapitulate H2A.Z identity, a combination of these nine unique regions must be required. Having established that the M6 region was both necessary and sufficient for H2A.Z’s binding to SWR1-C, we created a series of combination hybrids that contained the H2A.Z M6 region, plus one of the eight remaining H2A.Z regions, placed in the H2A protein background. After being transformed into the *htz1Δ* mutant, each hybrid again showed similar protein levels as the H2A.Z construct, except for a slight decrease in the H2A-H2A.Z[M6,N] and H2A-H2A.Z[M6,L1] constructs (S4A Fig). Growth assays comparing these hybrids identified two regions that were able to confer H2A.Z identity when combined with M6: the C-terminal tail and G4 (Figs 4A and S4B). While the *H2A-H2A*.*Z[M6*,*C]* mutant had improved growth over the *H2A-H2A*.*Z[M6]* mutant, the combination of the G4 and M6 regions (*H2A-H2A*.*Z[M6*,*G4]*) fully rescued *htz1Δ* mutant phenotypes. To more carefully quantify the G4 region’s affect on cell growth we performed liquid growth curves in the indicated concentration of formamide, which showed essentially identical growth patterns for strains containing the *H2A-H2A*.*Z[M6*,*G4]* mutant and the *H2A*.*Z* mutant (Fig 4B). In addition, the *H2A-H2A*.*Z[M6*,*G4]* mutant had a significant improvement in growth over the *H2A-H2A*.*Z[M6]* mutant, which was rather surprising given the G4 region only contains two amino acid changes from H2A to H2A.Z (R79K and N81L).

To gain a better understanding of the importance of K79 and L81 in H2A.Z identity, we compared the primary protein sequences of several H2A.Z homologs found in eukaryotic organisms. We found that both K79 and L81, which are located at the C-terminal proximal end of the α2-helix, are highly conserved in H2A.Z homologs and are consistently different from H2A (Fig 4C). Hybrid constructs containing the M6 region with either K79 or L81 showed that the phenotypes of the *H2A-H2A*.*Z[M6*,*G4]* mutant were primarily driven by L81 (Figs 4D and S4C). However, the *H2A-H2A*.*Z[M6*,*L81]* mutant did not fully recapitulate the phenotypes of the *H2A-H2A*.*Z[M6*,*G4]* mutant, indicating that both amino acids were required for the M6 and G4 regions to fully confer H2A.Z identity.

### Hybrid histone constructs did not recapitulate H2A.Z-specific enrichment at promoter regions despite having H2A.Z-like chromatin association

**Fig 5:**
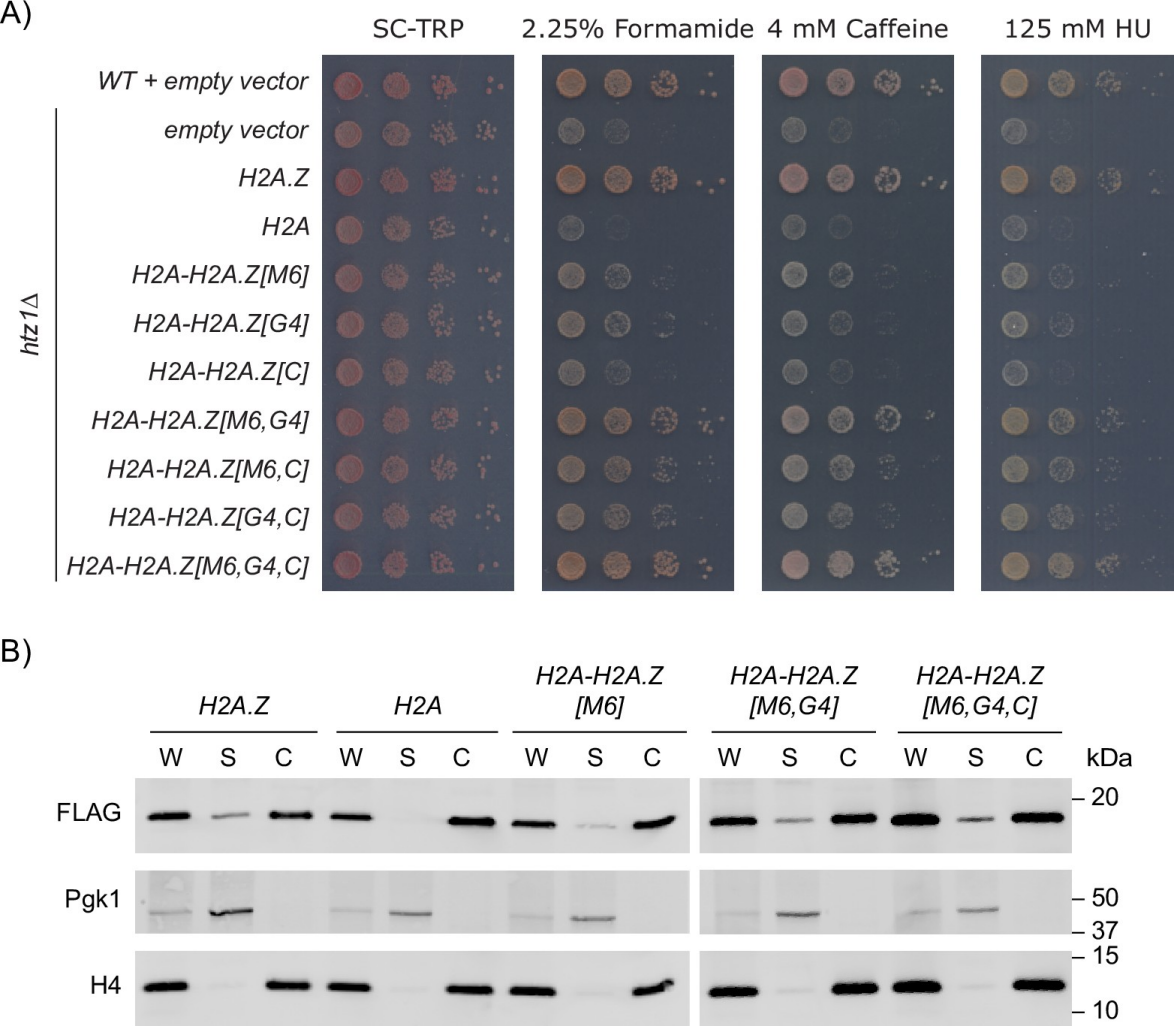
The M6 and G4 regions were sufficient to confer H2A.Z-specific bulk chromatin association. (A) In combination with G4 or the M6 region, the C-terminal tail conferred H2A.Z-specific growth phenotypes. However, the M6 and G4 regions were required to fully recapitulate H2A.Z identity. The majority of mutants here were previously shown in Fig 4A. Cells expressing the indicated hybrid constructs were 10-fold serially diluted, spotted onto SC-TRP media with the indicated concentrations of formamide, caffeine, and hydroxyurea and grown for 3 days. (B) All hybrid constructs associated with chromatin, however only the H2A-H2A.Z[M6,G4] and H2A-H2A.Z[M6,G4,C] constructs were present at similar levels in the soluble fraction as H2A.Z. Whole-cell extracts (W) were separated into chromatin (C) and soluble (S) (non-chromatin) fractions and analyzed by immunoblotting. FLAG antibodies detected the hybrid constructs, while H4 and Pgk1 were used as controls for the chromatin and soluble fractions, respectively.

As the *H2A-H2A*.*Z[M6*,*G4]* mutant rescued the growth phenotypes of the *htz1Δ* mutant in the tested stress conditions, it was formally possible that the M6 and G4 regions would be sufficient to recapitulate all aspects of H2A.Z identity. However as the *H2A-H2A*.*Z[M6*,*C]* mutant also improved growth over the *H2A-H2A*.*Z[M6]* mutant (Fig 4A), we speculated that a combination of all three regions may be required to recapitulate specific molecular aspects of H2A.Z function. To assess this possibility we created a triple hybrid containing the H2A.Z M6, G4, and C-terminal regions (*H2A-H2A*.*Z[M6*,*G4*,*C]*) which when placed in a *htz1Δ* background had similar growth phenotypes and protein abundance as the wild-type H2A.Z (Figs 5A and S5A and S5B).

To test the possibility that the differences in growth phenotypes between the *H2A-H2A*.*Z[M6]* mutant and the *H2A-H2A*.*Z[M6*,*G4]* and *H2A-H2A*.*Z[M6*,*G4*,*C]* mutants were the result of variation in chromatin association, we next determined if these hybrid constructs were incorporated into chromatin in a similar manner as wild-type H2A.Z. Bulk cellular fractionation assays revealed that, similar to H2A.Z, while the hybrid constructs were present predominantly in the chromatin fractions, appreciable levels were found in the soluble non-chromatin bound fraction (Figs 5B and S5C). In contrast, in the same experimental conditions the H2A construct was exclusively present in the chromatin fraction, consistent with previous work [53]. While in comparison to H2A the H2A-H2A.Z[M6] hybrid had increased levels in the non-chromatin supernatant fraction, only the H2A-H2A.Z[M6,G4] and H2A-H2A.Z[M6,G4,C] constructs fully recapitulated the levels of H2A.Z chromatin association.

**Fig 6:**
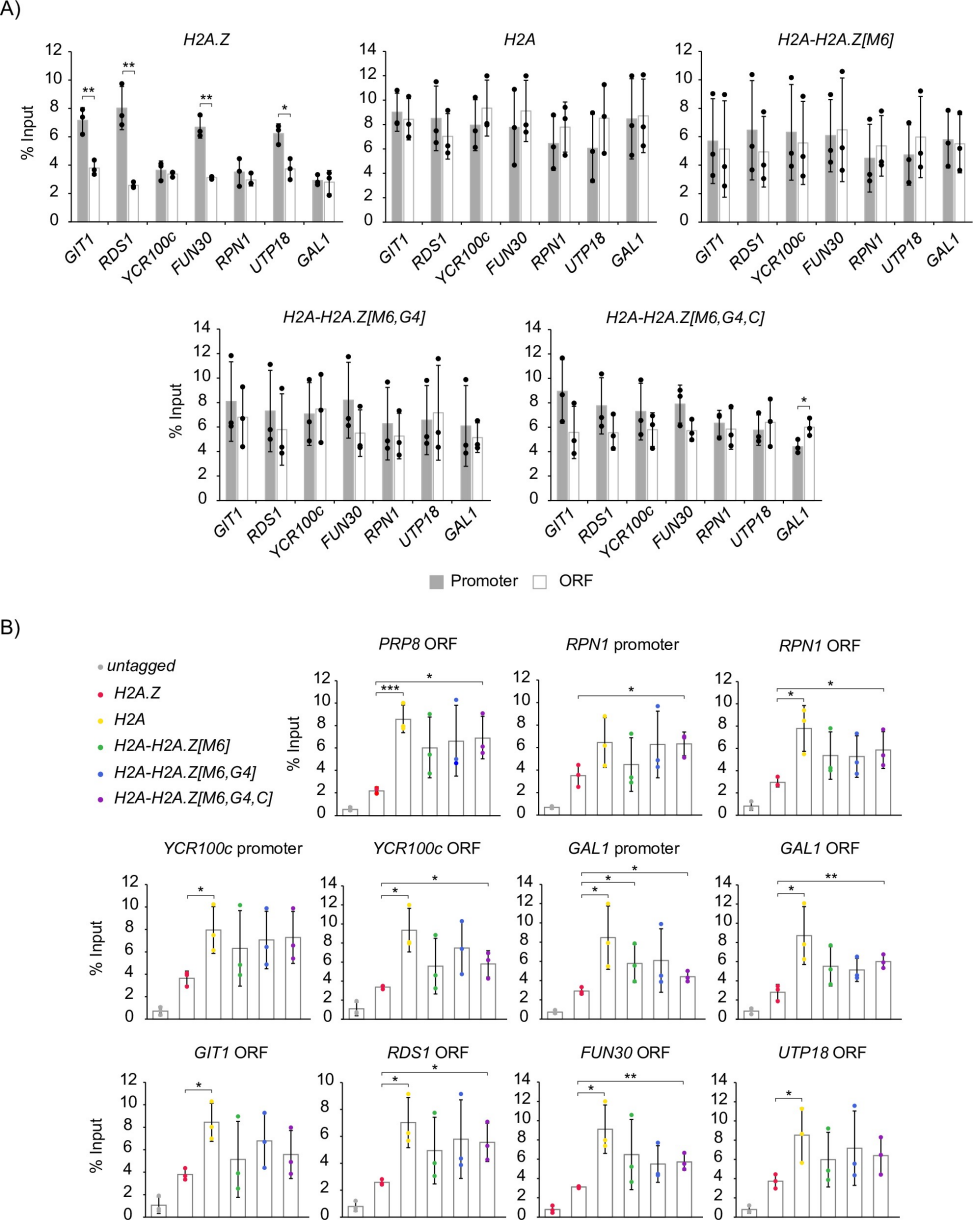
H2A-H2A.Z hybrid constructs were incorporated into chromatin but did not recapitulate H2A.Z-specific patterns of enrichment at gene promoters. (A) Unlike H2A.Z, the H2A-H2A.Z[M6], H2A-H2A.Z[M6,G4], and H2A-H2A.Z[M6,G4,C] constructs were not significantly enriched at promoters in comparison to their enrichment at respective gene ORFs. (B) At loci with relatively lower levels of H2A.Z, the H2A and H2A-H2A.Z[M6,G4,C] constructs had significantly increased enrichment in comparison to H2A.Z. FLAG-tagged hybrid enrichment levels determined by ChIP-qPCR for three replicates were normalized to their respective inputs. Asterisks indicate all the significant comparisons determined by unpaired two-tailed Student’s *t*-tests. * = p-value <0.05, ** = p-value <0.01, *** = p-value <0.001. All constructs were significantly enriched over the untagged control (p-value <0.05). All other unlabelled comparisons had a p-value > 0.05.

While H2A is uniformly distributed in chromatin, H2A.Z is primarily enriched in nucleosomes at gene promoters [34–39]. To test whether the H2A-H2A.Z[M6], H2A-H2A.Z[M6,G4], or H2A-H2A.Z[M6,G4,C] constructs were also selectively enriched at promoter regions, we compared the enrichment of each hybrid construct at both the promoter and 3’ end of the ORF of genes containing variable levels of H2A.Z by ChIP-qPCR (Figs 6A and S6A). Surprisingly, all three hybrid constructs showed overall patterns of enrichment more similar to H2A than H2A.Z. Consistent with previous work [65], H2A.Z was significantly enriched at the promoters of *GIT1*, *RDS1*, *FUN30*, and *UTP18* in comparison to loci in the ORF of each respective gene, while H2A was evenly enriched at both promoter and ORF locations. While H2A-H2A.Z[M6,G4,C] construct had a slight trend of increased enrichment at the promoters of a few genes in comparison to their respective ORF loci, like H2A, these comparisons were not statistically significant. Furthermore, the H2A-H2A.Z[M6,G4,C] construct had significantly increased enrichment over H2A.Z at several ORF and promoter regions (Fig 6B). Therefore while the H2A-H2A.Z[M6,G4] and H2A-H2A.Z[M6,G4,C] constructs had similar bulk chromatin association as H2A.Z, the M6, G4, and C-terminal regions were not sufficient to confer H2A.Z’s selective enrichment of promoter regions onto H2A.

### Three unique H2A.Z regions were sufficient to restore the mRNA levels of H2A.Z dependent genes

**Fig 7:**
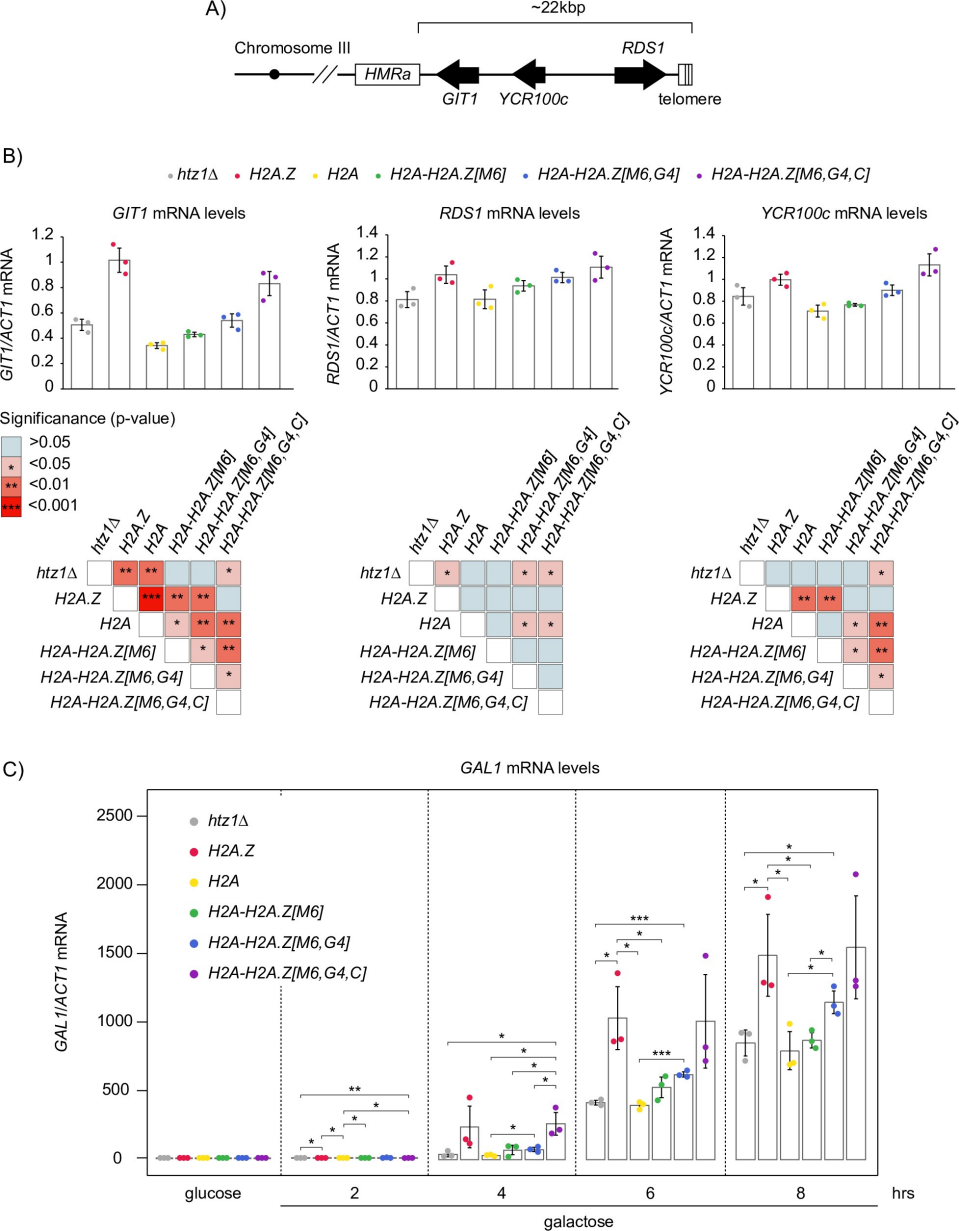
A combination of the three unique H2A.Z regions, M6, G4 and C-terminal tail, were sufficient to restore the mRNA levels of heterochromatin-proximal genes and *GAL1* after derepression. (A) Schematic representation of H2A.Z-dependent genes at the boundaries of the *HMRa* locus and the right telomere of chromosome III. Arrows represent open reading frames (ORFs) and point in the direction of transcription. (B) Expression defects observed in the *htz1Δ* mutant were gradually improved by the stepwise addition of the H2A.Z M6, G4, and C-terminal regions. RT-qPCR analysis of heterochromatin-proximal genes mRNA levels from three replicates were normalized to *ACT1* mRNA levels. Matrices of significant comparisons for *GIT1*, *RDS1*, and *YCR100c* were determined by unpaired two-tailed Student’s *t*-tests. (C) RT-qPCR analysis of *GAL1* mRNA levels performed on the indicated mutants, which were grown long-term in SC-TRP-glucose (2%) prior to being transferred to SC-TRP-galactose (2%) and collected at 2-hour intervals. During *GAL1* derepression, the addition of the M6, G4, and C-terminal regions incrementally increased *GAL1* expression, were the *H2A-H2A*.*Z[M6*,*G4*,*C]* mutant showed comparable levels to the *H2A*.*Z* mutant. *GAL1* mRNA levels were normalized to *ACT1*. Error bars indicate the standard deviation between the three replicates. Significance comparisons determined by unpaired two-tailed Student’s *t*-tests are indicated: * = p-value <0.05, ** = p-value <0.01, *** = p-value <0.001. All unlabelled comparisons within each time point had a p-value >0.05.

Curiously, while not having selective chromatin localization like H2A.Z, both the *H2A-H2A*.*Z[M6*,*G5]* and the *H2A-H2A*.*Z[M6*,*G5*,*C]* mutants had comparable growth phenotypes to wild-type, which suggested that G4 and C-terminal regions may still be important for the expression of H2A.Z-dependent genes. Using RT-qPCR we found that only the *H2A-H2A*.*Z[M6*,*G4*,*C]* mutant had comparable mRNA levels to the *H2A*.*Z* mutant for the heterochromatin-proximal genes *GIT1*, *RDS1*, and *YCR100c* (Fig 7A and 7B). Consistent with previous reports [53,66], all three genes showed a decrease in mRNA levels in the *htz1Δ* mutant. Furthermore, the *H2A* mutant had significantly reduced levels of mRNA for *RDS1* and *YCR100c* in comparison to the *H2A*.*Z* mutant. Addition of the H2A.Z M6 region slightly increased mRNA levels over the *H2A* mutant, while the *H2A-H2A*.*Z[M6*,*G4]* mutant had increased mRNA levels in comparison to the *H2A-H2A*.*Z[M6]* mutant. Therefore, while the M6, G4, and the C-terminal region each contributed to H2A.Z’s role in gene expression, only the combination of all three regions was sufficient to match the mRNA levels of the *H2A*.*Z* mutant and recapitulate H2A.Z function. However, there is a possibility that transcription defects observed for the *htz1Δ* mutant may not be entirely caused by the loss of H2A.Z-specific function. Swr1 hyper-accumulates at promoters in the absence of H2A.Z [67] and the *htz1Δswr1Δ* mutant partially alleviates *htz1Δ* mutant growth defects [31,32] (S7A Fig). This suggested that the presence of SWR1-C missing its H2A.Z substrate (apo-SWR1-C) could be affecting the transcription of these genes in the *htz1Δ* mutant. As substrates of SWR1-C, our hybrid constructs could then be simply alleviating apo-SWR1-C mischief at heterochromatin boundaries instead of recapitulating H2A.Z-specific function in transcription regulation. To address this important caveat, we performed RT-qPCR at *GIT1*, *RDS1*, and *YCR100c* for *htz1Δ*, *swr1Δ*, *htz1Δ swr1Δ* mutants (S7B Fig). The *htz1Δswr1Δ* mutant had comparable levels of mRNA to the *htz1Δ* mutant for each of these genes, which suggested that the decrease in expression for the *htz1Δ* mutant was primarily due to the loss of H2A.Z function in gene expression and not from the presence of apo-SWR1-C.

The expression of several inducible genes has been previously found to occur at significantly slower rates in a *htz1Δ* mutant than in wild-type cells [31,46–48]. Given that both the *H2A-H2A*.*Z[M6*,*G4]* and *H2A-H2A*.*Z[M6*,*G4*,*C]* mutants rescued the growth defects of the *htz1Δ* mutant when grown in 2% galactose (S8 Fig), we hypothesized that M6, G4, and C-terminal tail regions could also confer H2A.Z-specific expression for *GAL1* after derepression. We switched cells from media containing 2% glucose (which represses *GAL1* expression) to media with 2% galactose (which induces *GAL1*) and monitored *GAL1* mRNA levels before and after changing media. Similar to our results for the heterochromatin-proximal genes, the *H2A-H2A*.*Z[M6*,*G4*,*C]* mutant recapitulated the *H2A*.*Z* mutants *GAL1* mRNA levels for every time point (Fig 7C). Consistent with previous work, the *htz1Δ* mutant showed a slower increase in *GAL1* mRNA levels in comparison to the *H2A*.*Z* mutant [31,46,53]. Furthermore, both the *H2A* and *H2A-H2A*.*Z[M6]* mutants had comparable mRNA levels to the *htz1Δ* mutant throughout the time course. The *H2A-H2A*.*Z[M6*,*G4]* mutant however significantly increased levels of *GAL1* mRNA compared to the *H2A* mutant. Together with the results from the heterochromatin boundary genes, our results suggested that the M6, G4, and C-terminal tail regions were sufficient for the expression of H2A.Z-dependent genes.

## Discussion

**Fig 8:**
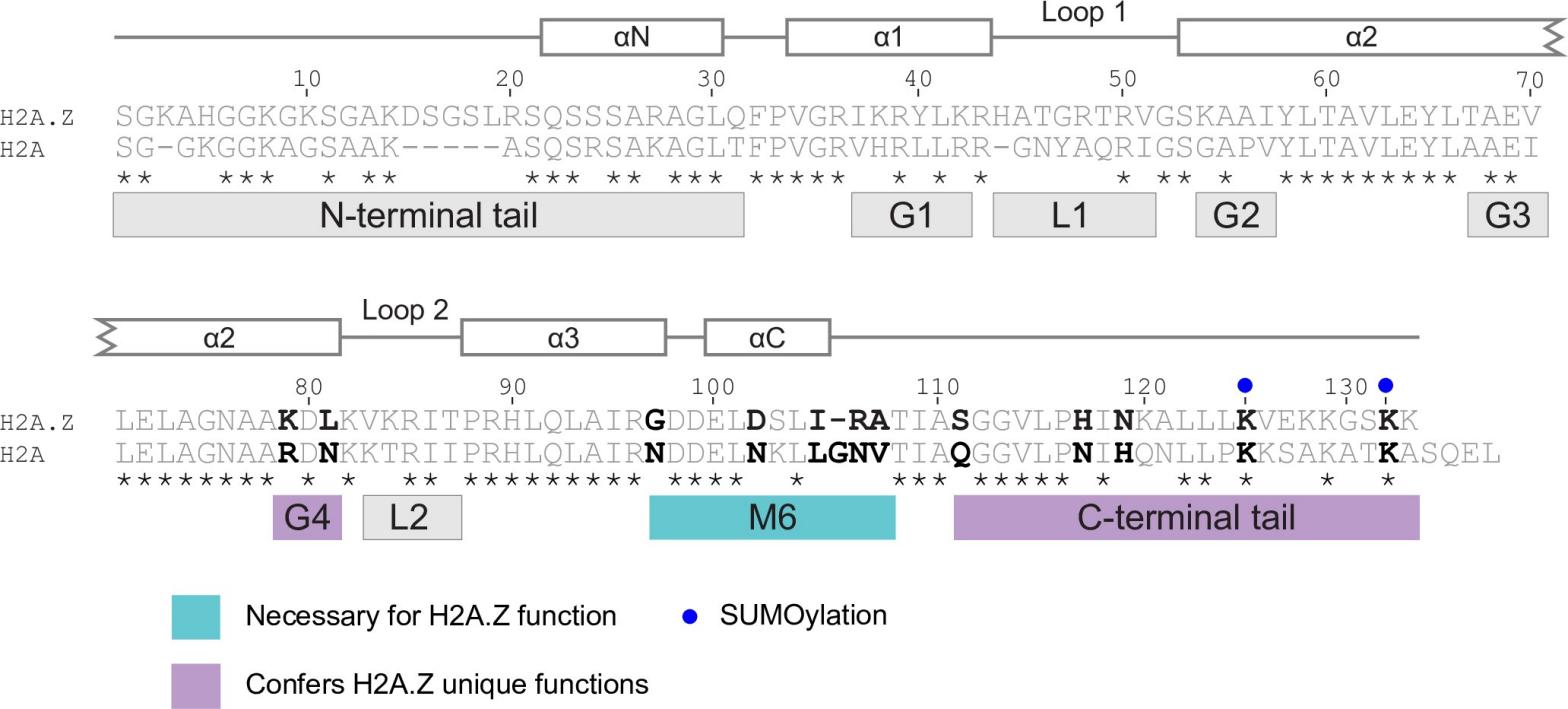
The M6, G4, and C-terminal regions of H2A.Z contributed to its unique function and distinguished it from H2A. Schematic of H2A.Z and H2A proteins highlighting the M6, G4, and C-terminal regions as key contributors to H2A.Z unique identity. The M6 region is necessary for H2A.Z function, while the G4 and C-terminal regions confer H2A.Z unique functions when combined with M6. Within these regions, amino acids that deviate between H2A.Z and H2A and that have been individually implicated in H2A.Z activities, by this study or previous literature, are highlighted in bold.

Determining what makes a histone variant a variant is a fundamental question in chromatin biology. While characterizing the amino acids required for H2A.Z function has been the focus of several previous studies, determining which regions are actually sufficient to confer histone variant identity, and thus responsible for distinguishing H2A.Z from H2A, have remained unclear. In this study, we used hybrid constructs based on replicative histone H2A to systematically analyze all of the divergent regions between H2A.Z and H2A. Here we report that the M6 region, the C-terminal tail, and the G4 region in the α2-helix each contributed to H2A.Z’s unique identity from H2A (Fig 8). While combining the M6 and G4 regions was sufficient for rescuing *htz1Δ* growth phenotypes, only the combination of the three unique H2A.Z regions (K79 and L81, M6, C-terminal tail) were sufficient for expression of H2A.Z-dependent heterochromatin-proximal genes and *GAL1* after derepression.

Here we report that while the M6 region was not sufficient to fully recapitulate H2A.Z identity, it was required for H2A.Z-specific function. We found that replacing the H2A.Z M6 region with the corresponding H2A residues resulted in growth defects in comparison to wild-type H2A.Z. Therefore, the seven amino acid differences between H2A and H2A.Z M6 regions must be required for H2A.Z-specific growth phenotypes (Fig 8). We confirmed previous reports which found that the H2A.Z M6 region is required for H2A.Z to interact with SWR1-C [61], which is unsurprising given that H2A.Z M6 region directly binds two of the complexes subunits, Swr1 and Swc2 [63,64]. Furthermore, we demonstrated that the H2A.Z M6 region was sufficient to fully recapitulate interaction with SWR1-C subunits (Fig 3A). This is consistent with work that compared crystal structures of H2A.Z-H2B dimers bound to Swr1 and found that a H2A hybrid construct containing the H2A.Z M6 residues I105-A107, had binding affinities for Swr1 that were similar to wild-type [63]. However, an additional structural study found that T87 (found in the L2 region in this study), D102 (in the M6 region) and the lack of a glycine in the H2A.Z M6 region, are important for differentiating H2A and H2A.Z’s binding affinity with Swc2 [64]. Despite this, we found that the H2A-H2A.Z[M6] construct had similar levels of association with Swc2 as the wild-type, indicating that only the M6 residues were necessary for association. Further supporting the M6 regions role as a binding motif, we also report that interchanging the H2A and H2A.Z M6 regions affected association with the histone chaperones, Chz1 and Nap1 (Fig 3B and 3C). This is consistent with previous work which has implicated G97 and the absence of a glycine in M6, as defining features in H2A.Z’s specific interaction with histone chaperones in yeast and mammals [27,68].

In this study we identified the two residues in the G4 region as key contributors to H2A.Z’s unique identity from H2A (Fig 8). The combination of K79 and L81 with the H2A.Z M6 region resulted in a complete rescue of *htz1Δ* growth phenotypes, an outcome not observed for any of the other regions when combined with M6 (Fig 4A). We also noted that the majority of G4’s impact on growth phenotypes was driven by L81. These data extend and contextualize previous results showing that a substitution of L81 with alanine results in a slight sensitivity to methyl methanesulfonate (MMS), while a K79 mutation does not [60]. This is largely unsurprising given that L81 (non-polar) has substantial differences in biochemical properties from its H2A counterpart (asparagine, a polar amino acid) while K79 has relatively similar properties to its H2A counterpart (arginine). Relatively little is known about the specific mechanistic or structural roles of K79 or L81 in H2A.Z function. There are currently no confirmed post-translational modifications for these amino acids in yeast, however tandem mass spectrometry indicates that the homologous lysine of H2A.Z K79 in humans (K74) is ubiquitinated [69]. It is possible that G4’s location just prior to the unstructured L2 loop region of the histone fold may be of significance given that the L2 region acts together with the H2B L1 loop to create a DNA-binding motif in the nucleosome [70]. Even small differences in the biochemical properties of adjacent amino acids could result in changes to the L2 loop positioning or flexibility. In humans, for example, the L1 loop regions of two isoforms of H2A.Z (H2A.Z.1 and H2A.Z.2) have identical amino-acid sequences, but crystal structures of nucleosomes containing either isoform indicate that these flexible regions have distinct structures that are at least partially caused by a single amino acid difference immediately prior to the L1 loop region (T38) [58]. We therefore propose that K79 and L81 may influence the flexibility or structure of the L2 loop region in yeast in a similar manner.

The large diversity of C-terminal tails is a key characteristic of the H2A family of histones and is thought to contribute to the distinct functions of many H2A variants, including H2A.Z [4,71]. Here we report that, in combination with the M6 and G4 regions, the C-terminal tail conferred H2A.Z driven gene expression for genes located at a heterochromatin-boundary as well as *GAL1* after derepression (Figs 7 and 8). This is consistent with previous work which has implicated the C-terminal tail as being important for wild-type transcription of both inducible genes and heterochromatin-boundary genes [46,47,53]. Despite this, our growth assay results for the *H2A*.*Z-H2A[C]* mutant suggested that the C-terminal tail can be functionally replaced with the H2A counterpart, which is consistent with previously published findings (Fig 2C) [53]. However, given that our results indicate that growth assay phenotypes do not necessarily equal complete wild-type function, it is possible that replacing the H2A.Z C-terminal tail with the H2A counter part could still result in expression defects for certain genes. In addition to having a role in transcription, together with M6, the H2A.Z C-terminal tail has been repeatedly shown to play an important role in H2A.Z incorporation and retention in chromatin [50,53,72,73]. The crystal structure shows that the C-terminal tail is anchored in the nucleosome, at least in part through hydrophobic interactions of side chains and backbone residues with histone H3 [50]. Truncations of this region result in the complete loss of H2A.Z in chromatin, and individual mutations of the H2A.Z specific residues S111, H117, and N119, also result in defects in chromatin association [53,72]. Finally, the H2A.Z C-terminal tail may also play a role in DNA damage repair. SUMOylation of K125 and K132 in the H2A.Z C-terminal tail in yeast is required for the recruitment of double strand breaks to the nuclear periphery [74]. As SUMO modifications have been shown to facilitate the exchange of H2A.Z.2 at DNA damage sites in human cells, a role in DNA damage repair may in fact be a conserved function of the H2A.Z C-terminal tail [75].

Throughout this study we used a broad variety of established *htz1Δ* phenotypes to determine if various amino acid regions are able to confer H2A.Z-specific functions when inserted into a H2A backbone. However previous studies have shown that mischief caused by the presence of apo-SWR1-C may be responsible for many of the defects observed in *htz1Δ* mutants [31–33]. Given that the presence of the H2A.Z M6 region was sufficient for wild-type levels of association with SWR1-C subunits (Fig 3A), it is possible that the hybrids containing the M6 region were simply recapitulating H2A.Z’s ability to alleviate apo-SWR1-C mischief. However, our results suggested that the hybrids did confer H2A.Z function in chromatin in addition to counteracting apo-SWR1-C. Specifically, and consistent with previous work, we showed that both the *swr1Δ* and *htz1Δswr1Δ* mutants grew better than the *htz1Δ* mutant, but still had minor grow defects in comparison to wild-type (S7A Fig) [31–33]. As this slow growth phenotype cannot be attributed to apo-SWR1-C, it likely represents defects caused by H2A.Z’s absence from chromatin. Therefore, since both the *H2A-H2A*.*Z[M6*,*G4]* and *H2A-H2A*.*Z[M6*,*G4*,*C]* mutants grew similar to wild-type, these regions likely contributed to H2A.Z-dependent functions in chromatin. Furthermore, the *htz1Δswr1Δ* mutant had similar levels of mRNA for the heterochromatin-boundary genes as the *htz1Δ* mutant, which suggested the stepwise recapitulation of expression observed in the hybrid mutants was not caused the alleviation of apo-SWR1-C mischief. However, previous work has shown that the *GAL1* expression defect in the *htz1Δ* mutant is partially repressed by the deletion of Swr1 [31], which suggests the impact of apo-SWR1-C on expression is gene dependent. Given the complexity of the circuitry between H2A.Z and SWR1-C, determining exactly what fraction of the *htz1Δ* mutant phenotype is caused directly by the of loss of H2A.Z function in chromatin is likely unattainable; however our results suggest that, at least in part, the M6, G4, and the C-terminal regions do confer H2A.Z function beyond alleviating apo-SWR1-C mischief.

During our examination of the enrichment of the H2A-H2A.Z hybrid constructs at gene promoters and their respective ORF regions we found that the addition of the M6, G4, and C-terminal tail regions were not sufficient to confer H2A.Z’s selective localization (Fig 6A and 6B). Given that H2A.Z’s distinct pattern of enrichment along chromatin is one of the definitive features differentiating between H2A and H2A.Z [3,5,7], we were surprised that hybrids lacking H2A.Z’s-specific localization still recapitulated H2A.Z dependent growth and gene expression (Figs 5A and 7). There is no doubt that H2A.Z-specific enrichment is necessary for many aspects of H2A.Z biology, such as promoting H3K27me3 deposition at promoters in *Arabidopsis* [76], yet our results suggested that general incorporation of the histone variant into chromatin may be sufficient for some H2A.Z functions. For example, based on our RT-qPCR data, H2A.Z’s role in restricting the spread of heterochromatin into euchromatin [66,77–80] may not be dependent on H2A.Z-specific enrichment. In the absence of H2A.Z, the SIR protein complex spreads from the silent mating loci, and a subset of telomeres, into the ORFs of adjacent regions causing decreased expression of the affected genes, including *GIT1*, *YCR100c*, and *RDS1* [66,80]. While the specific mechanism by which H2A.Z contributes to blocking heterochromatin spread is still poorly understood, studies using purified nucleosome arrays have found that H2A.Z containing chromatin is inherently resistant to condensation *in vitro* [81,82]. Therefore, the presence of H2A.Z in chromatin, and not its specific location, may be sufficient to prevent heterochromatin spread in yeast. This would also explain why the *H2A-H2A*.*Z[M6*,*G4]* and *H2A-H2A*.*Z[M6*,*G4*,*C]* mutants recapitulated the *H2A*.*Z* mutants mRNA levels for *YCR100c* despite more closely resembling the enrichment of the H2A construct at the *YCR100c* promoter (Figs 6B and 7B). In addition, our growth assay results also suggested that H2A.Z function in resistance to genotoxic stress may not be completely dependent on its specific enrichment levels, given that the *H2A-H2A*.*Z[M6*,*G4]* and *H2A-H2A*.*Z[M6*,*G4*,*C]* mutants had wild-type growth phenotypes despite not recapitulating H2A.Z-specific enrichment at all tested loci. Ultimately genome-wide high-resolution enrichment and expression analysis of H2A.Z mutants with defects in localization may yield insights into how H2A.Z might regulate transcription distinct from its specific localization patterns.

By utilizing a hybrid gene approach, we systematically analyzed each region of H2A.Z that is unique from H2A. While a multitude of previous studies have identified amino acids required for H2A.Z function [53,59,60], our study design has enabled us to distinguish which of these regions confer H2A.Z unique identity. In addition, while the exact mechanism by which H2A.Z affects gene expression still remains poorly understood, our results narrow down the amino acids responsible for this function, hopefully informing future research on the topic. Altogether, this study identified K79 and L81 as a new region that contributes to H2A.Z identity while also adding to our understanding of two key regions in H2A.Z biology, M6 and the C-terminal tail. As many of the amino acids in these regions are highly conserved in the mammalian homologs of H2A and H2A.Z, there is a strong possibility that these regions also serve to define H2A.Z unique identity in humans.

## Materials and methods

### Yeast strains and plasmids

All strains used in this study, listed in Table 1, were made using standard yeast genetic techniques. Gene deletions and the integration of a vesicular stomatitis virus (VSV) tag [83] at the 3’ end of genes were achieved using the one step gene replacement method [84]. All double mutant stains were generated via mating and tetrad dissection.

**Table 1:** Yeast strains used in this study.

Strain	Genotype	Source
MKY5	*W303 MATα ade2Δ1 can1Δ100 his3Δ11 leu2Δ3*,*112 trp1Δ1 ura3Δ1 LYS2*	
MKY1144	MKY5, *htz1Δ*::*HYGMX*	[53]
MKY1145	MKY5, *SWC2–VSV*::*KAN*	[53]
MKY1190	MKY5, *htz1Δ*::*HYGMX*, *SWC2–VSV*::*KANMX*	This study
MKY1194	MKY5, *htz1Δ*::*HYGMX*, *SWC2–VSV*::*KANMX*, pMK148	This study
MKY1195	MKY5, *htz1Δ*::*HYGMX*, *SWC2–VSV*::*KANMX*, pMK149	This study
MKY1196	MKY5, *htz1Δ*::*HYGMX*, *SWC2–VSV*::*KANMX*, pMK418	This study
MKY2052	MKY5, *htz1Δ*::*HYGMX*, *SWC2–VSV*::*KANMX*, pMK670	This study
MKY2053	MKY5, *htz1Δ*::*HYGMX*, *SWC2–VSV*::*KANMX*, pMK538	This study
MKY1153	MKY5, *SWC3–VSV*::*KAN*	[53]
MKY1191	MKY5, *htz1Δ*::*HYGMX*, *SWC3–VSV*::*KANMX*	This study
MKY1198	MKY5, *htz1Δ*::*HYGMX*, *SWC3–VSV*::*KANMX*, pMK148	This study
MKY1199	MKY5, *htz1Δ*::*HYGMX*, *SWC3–VSV*::*KANMX*, pMK149	This study
MKY1200	MKY5, *htz1Δ*::*HYGMX*, *SWC3–VSV*::*KANMX*, pMK418	This study
MKY2054	MKY5, *htz1Δ*::*HYGMX*, *SWC3–VSV*::*KANMX*, pMK670	This study
MKY2055	MKY5, *htz1Δ*::*HYGMX*, *SWC3–VSV*::*KANMX*, pMK538	This study
MKY1161	MKY5, SWC4-VSV	[53]
MKY1192	MKY5, *htz1Δ*::*HYGMX*, *SWC4–VSV*::*KANMX*	This study
MKY2056	MKY5, *htz1Δ*::*HYGMX*, *SWC4–VSV*::*KANMX*, pMK148	This study
MKY2057	MKY5, *htz1Δ*::*HYGMX*, *SWC4–VSV*::*KANMX*, pMK149	This study
MKY2058	MKY5, *htz1Δ*::*HYGMX*, *SWC4–VSV*::*KANMX*, pMK418	This study
MKY2059	MKY5, *htz1Δ*::*HYGMX*, *SWC4–VSV*::*KANMX*, pMK670	This study
MKY2060	MKY5, *htz1Δ*::*HYGMX*, *SWC4–VSV*::*KANMX*, pMK538	This study
MKY1177	MKY5, *CHZ1–VSV*::*KANMX*	[53]
MKY1193	MKY5, *htz1Δ*::*HYGMX*, *CHZ1–VSV*::*KANMX*	This study
MKY2061	MKY5, *htz1Δ*::*HYGMX*, *CHZ1–VSV*::*KANMX*, pMK148	This study
MKY2062	MKY5, *htz1Δ*::*HYGMX*, *CHZ1–VSV*::*KANMX*, pMK149	This study
MKY2063	MKY5, *htz1Δ*::*HYGMX*, *CHZ1–VSV*::*KANMX*, pMK418	This study
MKY2064	MKY5, *htz1Δ*::*HYGMX*, *CHZ1–VSV*::*KANMX*, pMK670	This study
MKY2065	MKY5, *htz1Δ*::*HYGMX*, *CHZ1–VSV*::*KANMX*, pMK538	This study
MKY2066	MKY5, pRS314	This study
MKY1185	MKY5, *htz1Δ*::*HYGMX*, pRS314	[53]
MKY1186	MKY5, *htz1Δ*::*HYGMX*, pMK148	[53]
MKY1187	MKY5, *htz1Δ*::*HYGMX*, pMK149	[53]
MKY2067	MKY5, *htz1Δ*::*HYGMX*, pMK418	This study
MKY2068	MKY5, *htz1Δ*::*HYGMX*, pMK670	This study
MKY1471	MKY5, *htz1Δ*::*HYGMX*, pMK538	This study
MKY2069	MKY5, *htz1Δ*::*HYGMX*, pMK671	This study
MKY2070	MKY5, *htz1Δ*::*HYGMX*, pMK672	This study
MKY2071	MKY5, *htz1Δ*::*HYGMX*, pMK673	This study
MKY2072	MKY5, *htz1Δ*::*HYGMX*, pMK674	This study
MKY2073	MKY5, *htz1Δ*::*HYGMX*, pMK675	This study
MKY2074	MKY5, *htz1Δ*::*HYGMX*, pMK676	This study
MKY2075	MKY5, *htz1Δ*::*HYGMX*, pMK677	This study
MKY2076	MKY5, *htz1Δ*::*HYGMX*, pMK678	This study
MKY2077	MKY5, *htz1Δ*::*HYGMX*, pMK679	This study
MKY2078	MKY5, *htz1Δ*::*HYGMX*, pMK680	This study
MKY2079	MKY5, *htz1Δ*::*HYGMX*, pMK681	This study
MKY2080	MKY5, *htz1Δ*::*HYGMX*, pMK682	This study
MKY2081	MKY5, *htz1Δ*::*HYGMX*, pMK683	This study
MKY2082	MKY5, *htz1Δ*::*HYGMX*, pMK684	This study
MKY2083	MKY5, *htz1Δ*::*HYGMX*, pMK685	This study
MKY2084	MKY5, *htz1Δ*::*HYGMX*, pMK686	This study
MKY2085	MKY5, *htz1Δ*::*HYGMX*, pMK687	This study
MKY2086	MKY5, *htz1Δ*::*HYGMX*, pMK688	This study
MKY2087	MKY5, *htz1Δ*::*HYGMX*, pMK689	This study
MKY2088	MKY5, *htz1Δ*::*HYGMX*, pMK690	This study
MKY2089	MKY5, *htz1Δ*::*HYGMX*, pMK691	This study
MKY2090	MKY5, *htz1Δ*::*HYGMX*, pMK692	This study
MKY2091	MKY5, *htz1Δ*::*HYGMX*, pMK693	This study
MKY2092	MKY5, *htz1Δ*::*HYGMX*, pMK694	This study
MKY2093	MKY5, *htz1Δ*::*HYGMX*, pMK695	This study
MKY2094	MKY5, *htz1Δ*::*HYGMX*, pMK696	This study
MKY2095	MKY5, *htz1Δ*::*HYGMX*, pMK697	This study
MKY2096	MKY5, *htz1Δ*::*HYGMX*, pMK698	This study
MKY2097	MKY5, *swr1Δ*::*HIS*, pRS314	This study
MKY2098	MKY5, *htz1Δ*::*HYGMX*, *swr1Δ*::*HIS*, pRS314	This study

Table 2 lists all plasmids used in this study. Both the parent *H2A*.*Z* and *H2A* pRS314 plasmids were expressed from the *HTZ1* promoter and C-terminally tagged with *3xFLAG* [53]. These parent plasmids were used to generate each of the hybrid constructs using standard molecular techniques. The QuickChange site directed mutagenesis technique (Agilent) was adapted to replace both the N-terminal and C-terminal tail regions. Multiple rounds of site-directed mutagenesis were performed to replace the remaining regions (primers available upon request).

**Table 2:** Plasmids used in this study.

Plasmid	Relevant Genotype	Source
pRS314	*TRP1*, *CEN6/ARSH4*	[85]
pMK148	pRS314, *HTZ1*	[80]
pMK149	pRS314, *HTZ1-3×FLAG*::*KANMX*	[53]
pMK418	pRS314, *HTA1-3×FLAG*::*KANMX*	[53]
pMK670	pRS314, *HTZ1-HTA1[M6]-3×FLAG*::*KANMX*	This study
pMK538	pRS314, *HTA1-HTZ1[M6]-3×FLAG*::*KANMX*	This study
pMK671	pRS314, *HTA1-HTZ1[N]-3×FLAG*::*KANMX*	This study
pMK672	pRS314, *HTA1-HTZ1[C]-3×FLAG*::*KANMX*	This study
pMK673	pRS314, *HTA1-HTZ1[G1]-3×FLAG*::*KANMX*	This study
pMK674	pRS314, *HTA1-HTZ1[L1]-3×FLAG*::*KANMX*	This study
pMK675	pRS314, *HTA1-HTZ1[G2]-3×FLAG*::*KANMX*	This study
pMK676	pRS314, *HTA1-HTZ1[G3]-3×FLAG*::*KANMX*	This study
pMK677	pRS314, *HTA1-HTZ1[G4]-3×FLAG*::*KANMX*	This study
pMK678	pRS314, *HTA1-HTZ1[L2]-3×FLAG*::*KANMX*	This study
pMK679	pRS314, *HTZ1-HTA1[N]-3×FLAG*::*KANMX*	This study
pMK680	pRS314, *HTZ1-HTA1[C]-3×FLAG*::*KANMX*	This study
pMK681	pRS314, *HTZ1-HTA1[G1]-3×FLAG*::*KANMX*	This study
pMK682	pRS314, *HTZ1-HTA1[L2]-3×FLAG*::*KANMX*	This study
pMK683	pRS314, *HTZ1-HTA1[G2]-3×FLAG*::*KANMX*	This study
pMK684	pRS314, *HTZ1-HTA1[G3]-3×FLAG*::*KANMX*	This study
pMK685	pRS314, *HTZ1-HTA1[G4]-3×FLAG*::*KANMX*	This study
pMK686	pRS314, *HTZ1-HTA1[L2]-3×FLAG*::*KANMX*	This study
pMK687	pRS314, *HTA1-HTZ1[M6*,*N]-3×FLAG*::*KANMX*	This study
pMK688	pRS314, *HTA1-HTZ1[M6*,*C]-3×FLAG*::*KANMX*	This study
pMK689	pRS314, *HTA1-HTZ1[M6*,*G1]-3×FLAG*::*KANMX*	This study
pMK690	pRS314, *HTA1-HTZ1[M6*,*L1]-3×FLAG*::*KANMX*	This study
pMK691	pRS314, *HTA1-HTZ1[M6*,*G2]-3×FLAG*::*KANMX*	This study
pMK692	pRS314, *HTA1-HTZ1[M6*,*G3]-3×FLAG*::*KANMX*	This study
pMK693	pRS314, *HTA1-HTZ1[M6*,*G4]-3×FLAG*::*KANMX*	This study
pMK694	pRS314, *HTA1-HTZ1[M6*,*L2]-3×FLAG*::*KANMX*	This study
pMK695	pRS314, *HTA1-HTZ1[M6*,*K79]-3×FLAG*::*KANMX*	This study
pMK696	pRS314, *HTA1-HTZ1[M6*,*L81]-3×FLAG*::*KANMX*	This study
pMK697	pRS314, *HTA1-HTZ1[G4*,*C]-3×FLAG*::*KANMX*	This study
pHB698	pRS314, *HTA1-HTZ1[G4*,*M6*,*C]-3×FLAG*::*KANMX*	This study

### Protein extraction and protein blotting

Overnight cultures grown in synthetic complete media without tryptophan (SC-TRP) were diluted to 0.3 OD_600_ and grown to 1.0 OD_600_. Whole-cell extracts were prepared by glass bead lysis in the presence of trichloroacetic acid [86]. Immunoblotting was performed using anti-FLAG (Sigma), anti-HTZ1 (Active Motif) and anti-PGK1 (Life Technologies) antibodies. Immunoblots were scanned with the Odyssey Infrared Imaging System (Life Technologies) and analyzed using the Image Studio software (LI-COR). Three biological replicates were performed for each culture.

### Growth assays

Overnight cultures grown in SC-TRP were diluted to 0.5 OD_600_. Cells were then 10-fold serially diluted and spotted onto SC-TRP plates with or without the indicated amount of caffeine (Sigma), hydroxyurea (HU) (Sigma), and formamide (Sigma). Plates were incubated for three days at 30°C. Three biological replicates were performed for each culture.

### Small-scale interaction assays

Co-immunoprecipitation (IP) of protein complexes were performed in triplicate as previously described [23]. Briefly, overnight cultures were diluted to 0.3 OD_600_ in 50 mL of SC-TRP and grown to 1.0 OD_600_. Cells were harvested and resuspended in TAP-IP buffer (50 mM Tris [pH 7.8], 150 mM NaCl, 1.5 mM MgAc, 0.15% NP-40, 1 mM DTT, 10 mM NaPPi, 5 mM EGTA, 5 mM EDTA, 0.1 mM Na_3_VO_4_, 5 mM NaF, 1 μg/mL leupeptin, 2 μg/mL aprotinin, 1 mM PMSF). Cells were mechanically lysed with glass beads for 5 minutes using a bead beater (BioSpec Products). Lysates were incubated with 40 μL of FLAG M2 agarose beads (Sigma) for 1.5 hours, or 20 μL of VSV agarose beads (MBL) for 16 hours. The beads were washed once with TAP-IP buffer containing protease inhibitors and twice with TAP-IP buffer lacking protease inhibitors. The beads were resuspended and boiled in SDS-PAGE sample buffer. Samples were analyzed by using SDS-PAGE followed by western blotting. Captured proteins were detected with anti-FLAG M2 (Sigma), anti-VSV (Bethyl Laboratories), and anti-Nap1 (Santa Cruz Biotechnology). Bands were visualized using Odyssey Infrared Imaging System (LI-COR).

### Liquid growth curves

Overnight cultures were diluted to 0.1 OD_600_ and then grown to 0.5 OD_600_. Cultures were diluted to 0.1 OD_600_ in 200 μL SC-TRP media plus 1.5% formamide (Sigma). OD_600_ readings were measured every 30 minutes over a period of 35 hours in a TECAN M200 plate reader. Plates were shaken for 10 minutes before each reading. Strains were tested in three biological replicates per plate and the area under the curve (AUC) was calculated for each replicate.

### Chromatin association assays

Chromatin association assays were performed in triplicate as previously described [87]. Overnight cultures were diluted to 0.2 OD_600_ in 50 mL of SC-TRP and grown to 0.5 OD_600_. A total of 25 OD units were harvested, incubated in Pre-spheroplast Buffer (100 mM PIPES/KOH [pH 9.4], 10 mM DTT, 0.1% sodium azide) for 10 minutes at room temperature, and spheroplasted with 20 mg/mL Zymolyase-100T (Seikagaku Corporation) in Spheroplast Buffer (50 mM KPO_4_ [pH 7.5], 0.6 M sorbitol, 10 mM DTT) for 30 minutes at 37°C. The resulting spheroplasts were washed with Wash Buffer (50 mM HEPES/KOH [pH 7.5], 100 mM KCl, 2.5 mM MgCl_2_, 0.4 M sorbitol), resuspended in EB (50 mM HEPES/KOH [pH 7.5], 100 mM KCl, 2.5 mM MgCl_2_, 1 mM DTT, 1 mM PMSF, 1 μg/mL leupeptin, 2 μg/mL aprotinin, 1 mM PMSF) and lysed on ice with 0.1% Triton X-100. A portion of the resulting whole cell extracts (WCE) were saved, while the remaining lysate was centrifuged through EBSX (EB + 0.25% Triton X-100 and 30% sucrose) to separate the chromatin pellet and supernatant fractions. The WCE, pellet, and supernatant fractions were analysed using SDS-PAGE followed by immunoblotting with anti-FLAG M2 (Sigma), anti-H4 (Abcam), and anti-PGK1 (Sigma) antibodies. Bands were visualized using Odyssey Infrared Imaging System (LI-COR).

### ChIP-qPCR

Overnight cultures were diluted to 0.15 OD_600_ in 250 mL of SC-TRP media and grown to 0.5 OD_600_. The procedure for ChIP was adapted from a previous report [88]. In brief, cultures were cross-linked with 1% formaldehyde for 20 minutes. Chromatin was sonicated (10 cycles, 30 seconds on/off, high setting [Bioruptor;Diagenode, Sparta, NJ]) to yield an average DNA fragment of 500 bp. Sheared chromatin was incubated with anti-FLAG antibody (4.2 μL) (Sigma, F3165) coupled to 60 μL of Protein A Dynabeads (Invitrogen) for 3 hours. Following crosslinking reversal and DNA purification, both the immunoprecipitated and input DNA were analyzed by qPCR using a Rotor-Gene 6000 (Corbett Research) and PerfeCTa SYBR green FastMix (Quanta Biosciences). All samples were analyzed from three independent biological replicates and normalized to percent input (0.7%). Primers used are listed in Table 3.

**Table 3:** RT-qPCR and ChIP-qPCR primers.

Primer name	Forward sequence	Reverse sequence
*ACT1* RT-qPCR	TGTCCTTGTACTCTTCCGGT	CCGGCCAAATCGATTCTCAA
*GAL1* RT-qPCR	GGTGGTTGTACTGTTCACTTGGTTCC	TCATATAGACAGCTGCCCAATGCTG
*GIT1* RT-qPCR	ATCGGTTCTGTAGTAGGCG	TTACCAGTCCAGCCATTGG
*RDS1* RT-qPCR	AAGCCGTGAGATTGAAATGG	CTCCATCTGGCACAACAGAA
*YCR100c* RT-qPCR	CCAGATGGATCAGGCTCAAA	TCGATCGCATACAGGACACT
*PRP8* ORF	GGATGTATCCAGAGGCCAAT	AACCCGCGTATTAAGCCATA
*RPN1* Promoter	CGGATAGCTGCTCCTCTTCC	TGCCCATTGGTCTACATAAGGT
*RPN1* ORF	AAGTAGCCGCAGATCCATCG	AGGGTGGGGACATGAGTACA
*FUN30* Promoter	TGGTGGACACCCGACTATCT	ACGACGATGCTACCTTGGTG
*FUN30* ORF	GATGACGAATTGCCGCAGTC	TGGTGGAGATCACGTGTGTG
*GAL1* Promoter	GGGTAATTAATCAGCGAAGCGATG	TGCGCTAGAATTGAACTCAGGTAC
*GAL1* ORF	TCTTCTTCGGCCGCATTCAT	AAACAGAGGCAGCCTGATCC
*UTP18* Promoter	TTCATCTGGTGGAGGTACGC	CTGGTCCTGTTGTGGTATCGT
*UTP18* ORF	ACACCTTCTATCTTGCAGCCC	TGGACATGAGTCCACTCAAAGA
*GIT1* Promoter	TTCATGAATTTCCTTACTGGAC	GTTGACTAGTCACAAGAAACAG
*GIT1* ORF	CGACGCCTACTACAGAACCG	AGCAACCGCTGTTAGAGGTG
*RDS1* Promoter	TGTGCTATCTAAGAGGATGGTTCA	CAGCAGCCAATTTCATGTTC
*RDS1* ORF	TTGCTGAAGGTGATGCTGGT	ACGTTCGATTCACTCGCAGA
*YCR100c* Promoter	GCAAGGATTCTGACTTTACTGG	CTCGTTATGCCCGTCATCTT
*YCR100c* ORF	CCCTCCATGTTAGACCACCG	TGACAGGTTCTGTTGGCGAT

### RT-qPCR

Overnight yeast cultures were diluted to 0.15 OD_600_ in 40 mL of SC-TRP. Cells were grown to an OD_600_ of 0.5 and a total of 10 OD units were collected. RNA was extracted using the RNeasy Mini Kit (Qiagen) and converted to cDNA using the QuantiTect Reverse Transcription Kit (Qiagen). cDNA was analyzed using a Rotor-Gene 6000 (Corbett Research) and PerfeCTa SYBR green FastMix (Quanta Biosciences). Samples were analyzed from three independent biological replicates and normalized to *ACT1*. Primer sequences are listed in Table 3.

### Galactose derepression

The procedure for growth of yeast cultures for *GAL1* derepression was adapted from a previous report [31]. Overnight cultures were diluted to 0.1 OD_600_ in 175 mL in SC-TRP (glucose, 2%). Once cells had reached an OD_600_ of 0.45, 7.5 OD units were collected (time point “0”). The remaining cells were washed with sterile ddH_2_O and resuspended in 150 mL of SC-TRP containing 2% galactose. Samples were taken 2, 4, 6, and 8 hours after cells were switched to galactose media, with 7.5 OD units collected at each timepoint. RT-qPCR was carried out for three independent biological replicates as described above.

## Supporting information

S1 Fig H2A/H2A.Z hybrid constructs had similar levels of abundance as the H2A.Z construct. (A) Immunoblotting of whole-cell extracts showed that plasmid-based H2A.Z was slightly more abundant than endogenously expressed H2A.Z. H2A.Z abundance was assessed using anti-FLAG and anti-H2A.Z. (B) Immunoblotting of whole-cell extracts of the *H2A-H2A*.*Z* mutants and (C) the *H2A*.*Z-H2A* mutants indicated that each hybrid construct was present in comparable levels to the H2A.Z construct. Pgk1 was used as a loading control.
(TIF)

S2 Fig Growth assay biological replicates for Fig 2. (A) Replicate 2 and 3 for Fig 2B. (B) Replicate 2 and 3 for Fig 2C. Cells expressing the indicated hybrid constructs were 10-fold serially diluted, spotted onto SC-TRP media with the indicated concentrations of formamide, caffeine, and hydroxyurea and grown for 3 days.
(TIF)

S3 Fig Small-scale interaction assay biological replicates for Fig 3. (A) Replicate 2 and 3 of Fig 3A co-purification with VSV-tagged Swc2 and Swc3. (B) All biological replicates for Fig 3A co-purification with VSV-tagged Swc4. The lanes from replicates 1 and 2 were used to produce Fig 3. Bolded strains in replicate 1 highlight strains were loaded in different order from other replicates. “*” indicates there was a transfer issue in this lane requiring another image to confirm the results for the affected sample. (C) Replicate 2 and 3 of Fig 3B co-purifications with Nap1 and VSV-tagged Chz1. (D) Replicate 2 and 3 of Fig 3C Chz1-VSV reciprocal immunoprecipitation.
(TIF)

S4 Fig Protein abundance and biological replicates of H2A-H2A.Z combination hybrids from Fig 4. (A) Immunoblotting of whole-cell extracts indicated that all combination hybrid constructs were present in the mutants in similar levels as the H2A.Z construct. Pgk1 was used as a loading control. (B) Biological replicates of growth assays for Fig 4A. (C) Biological replicates of growth assays for Fig 4D. Cells expressing the indicated hybrid constructs were 10-fold serially diluted, spotted onto SC-TRP media with the indicated concentrations of formamide, caffeine, and hydroxyurea and grown for 3 days.
(TIF)

S5 Fig Protein abundance and biological replicates for Fig 5. (A) Protein levels of C-terminal hybrid constructs from Fig 5A were analyzed by immunoblotting of whole-cell extracts with an anti-FLAG antibody, with Pgk1 was used as a loading control. (B) Biological replicates of growth assays for Fig 5A. Cells expressing the indicated hybrid constructs were 10-fold serially diluted, spotted onto SC-TRP media with the indicated concentrations of formamide, caffeine, and hydroxyurea and grown for 3 days. (C) Biological replicates of chromatin association assay in Fig 5B. Whole-cell extracts (W) were separated into chromatin (C) and soluble (S) (non-chromatin) fractions and analyzed by immunoblotting. FLAG antibodies detected the hybrid constructs, while H4 and Pgk1 were used as controls for the chromatin and soluble fractions, respectively.
(TIF)

S6 Fig Enrichment of untagged construct at promoter and ORF loci. (A) FLAG-tagged hybrid enrichment levels for the untagged control determined by ChIP-qPCR for three replicates were normalized to their respective inputs. (B) Enrichment of all constructs at promoter loci that were relatively enriched for H2A.Z in comparison to the gene ORF (see Fig 6A). While all constructs were enriched over the untagged control (P > 0.05) all other comparisons were non-significant as determined by unpaired two-tailed Student’s *t*-tests.
(TIF)

S7 Fig H2A-H2A.Z hybrids conferred H2A.Z-specific function in addition to counteracting apo-SWR1-C mischief. (A) The *H2A-H2A*.*Z[M6*,*G4]* and *H2A-H2A*.*Z[M6*,*G4*,*C]* mutants in a *htz1Δ* background had improved growth in comparison to the *swr1Δ* or *swr1Δhtz1Δ* mutants and had similar growth phenotypes to the *H2A*.*Z* mutant. Cells expressing the indicated hybrid constructs were 10-fold serially diluted, spotted onto SC-TRP media with the indicated concentrations of formamide, caffeine, and hydroxyurea and grown for 3 days. (C) Decrease in mRNA levels in the *htz1Δ* mutant for heterochromatin-proximal genes was not caused by the presence of apo-SWR1-C. RT-qPCR analysis of heterochromatin-proximal genes mRNA levels from three replicates were normalized to *ACT1* mRNA levels. Error bars indicate the standard deviation between the three replicates. Significant comparisons determined by unpaired two-tailed Student’s *t*-tests are indicated: ** = p-value <0.01.
(TIF)

S8 Fig The *htz1Δ* mutant had a slight growth defect in 2% galactose media. Cells expressing the indicated hybrid constructs were 10-fold serially diluted, spotted onto SC-TRP media containing either 2%-glucose or 2%-galactose with 0.02 mg/mL ethidium bromide and grown for 3 days.
(TIF)
