# Supplementary figures and images for: What makes a histone variant a variant: Changing H2A to become H2A.Z
Source: PLoS Genet. 2021 Dec 6;17(12):e1009950. doi: 10.1371/journal.pgen.1009950 (PMC8675926; doi:10.1371/journal.pgen.1009950)

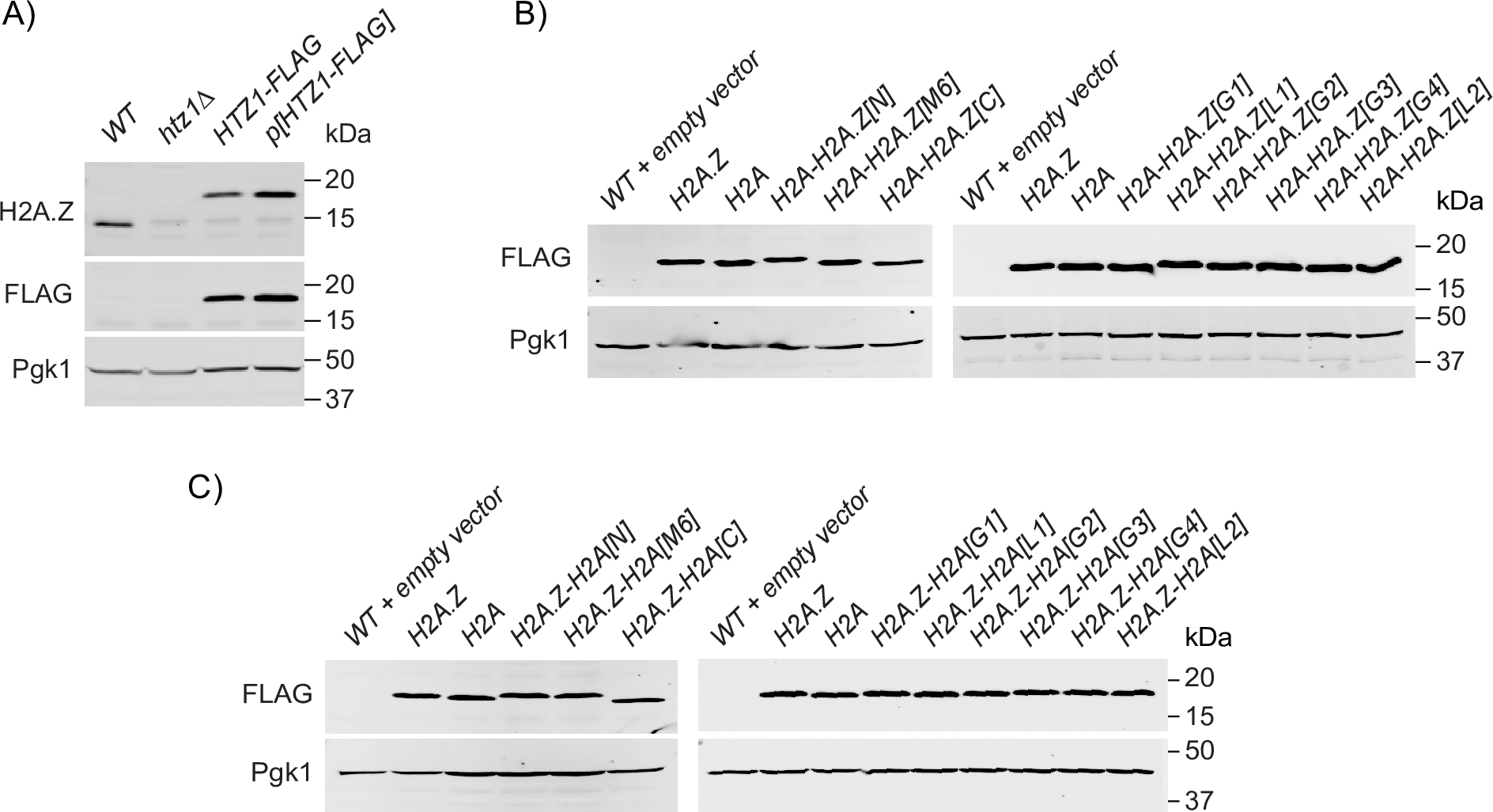

Supplement: S1 Fig — (A) Immunoblotting of whole-cell extracts showed that plasmid-based H2A.Z was slightly more abundant than endogenously expressed H2A.Z. H2A.Z abundance was assessed using anti-FLAG and anti-H2A.Z. (B) Immunoblotting of whole-cell extracts of the H2A-H2A.Z mutants and (C) the H2A.Z-H2A mutants indicated that each hybrid construct was present in comparable levels to the H2A.Z construct. Pgk1 was used as a loading control. (TIF) [file pgen.1009950.s001.tif]

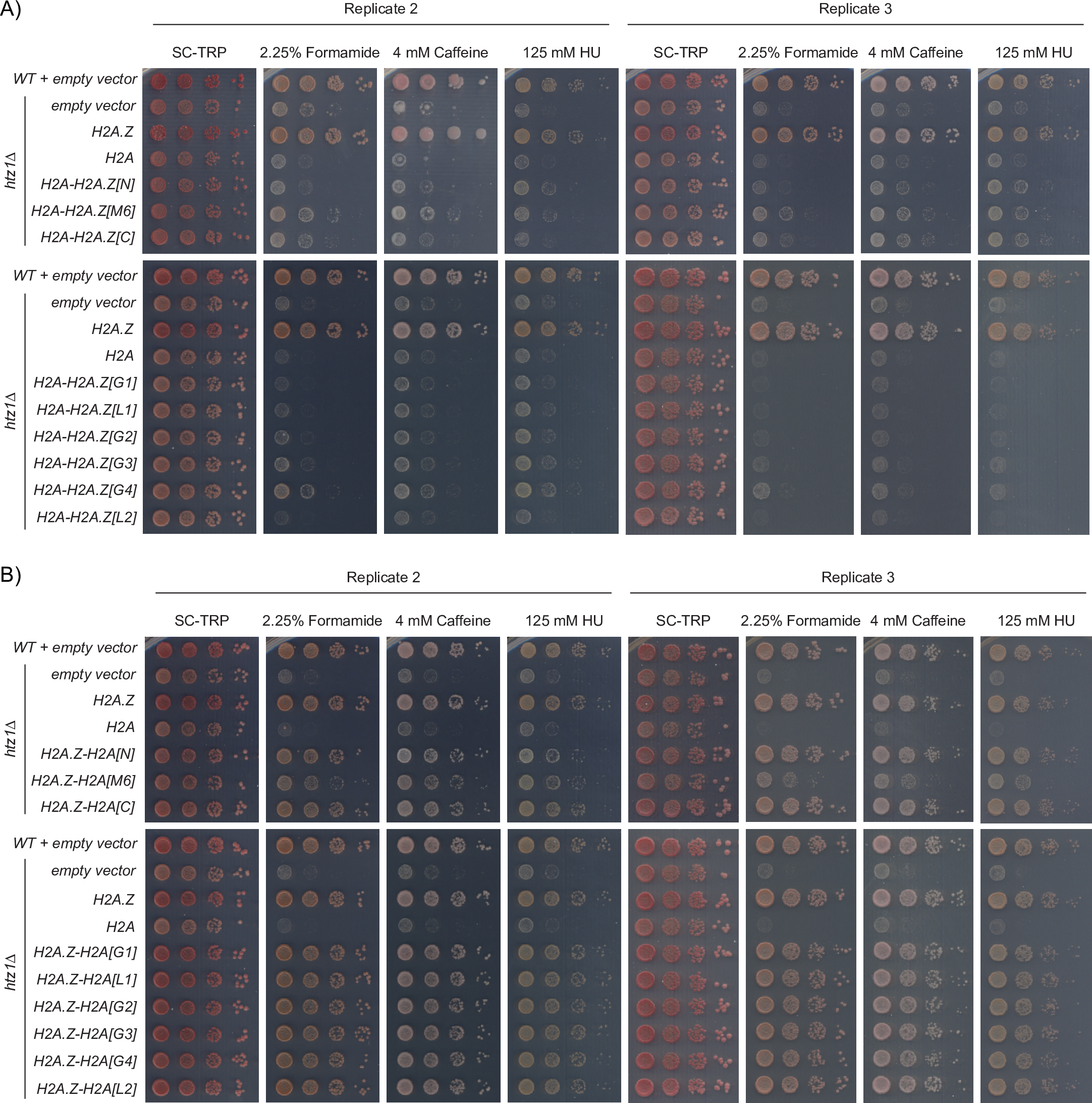

Supplement: S2 Fig — (A) Replicate 2 and 3 for Fig 2B. (B) Replicate 2 and 3 for Fig 2C. Cells expressing the indicated hybrid constructs were 10-fold serially diluted, spotted onto SC-TRP media with the indicated concentrations of formamide, caffeine, and hydroxyurea and grown for 3 days. (TIF) [file pgen.1009950.s002.tif]

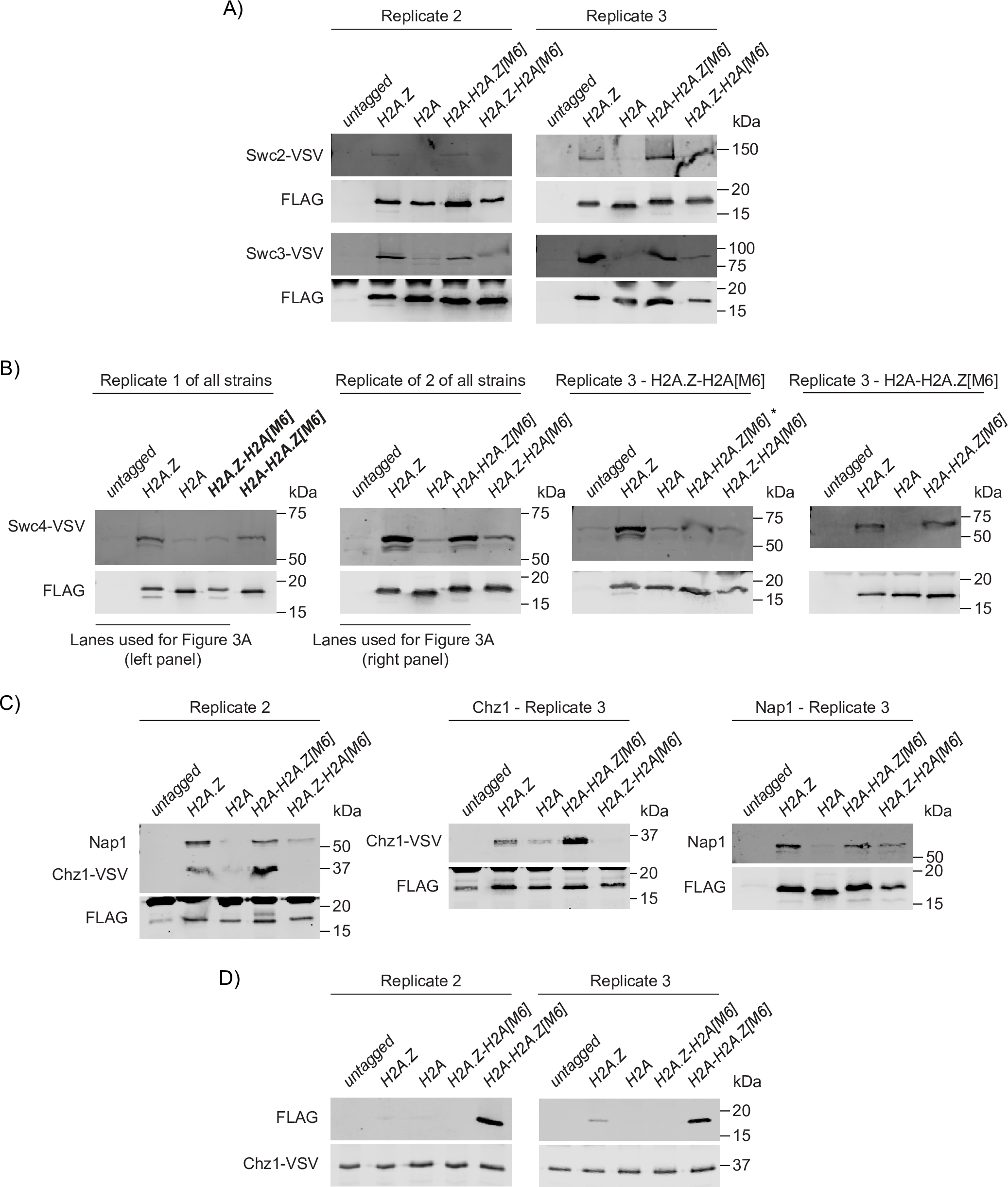

Supplement: S3 Fig — (A) Replicate 2 and 3 of Fig 3A co-purification with VSV-tagged Swc2 and Swc3. (B) All biological replicates for Fig 3A co-purification with VSV-tagged Swc4. The lanes from replicates 1 and 2 were used to produce Fig 3. Bolded strains in replicate 1 highlight strains were loaded in different order from other replicates. “*” indicates there was a transfer issue in this lane requiring another image to confirm the results for the affected sample. (C) Replicate 2 and 3 of Fig 3B co-purifications with Nap1 and VSV-tagged Chz1. (D) Replicate 2 and 3 of Fig 3C Chz1-VSV reciprocal immunoprecipitation. (TIF) [file pgen.1009950.s003.tif]

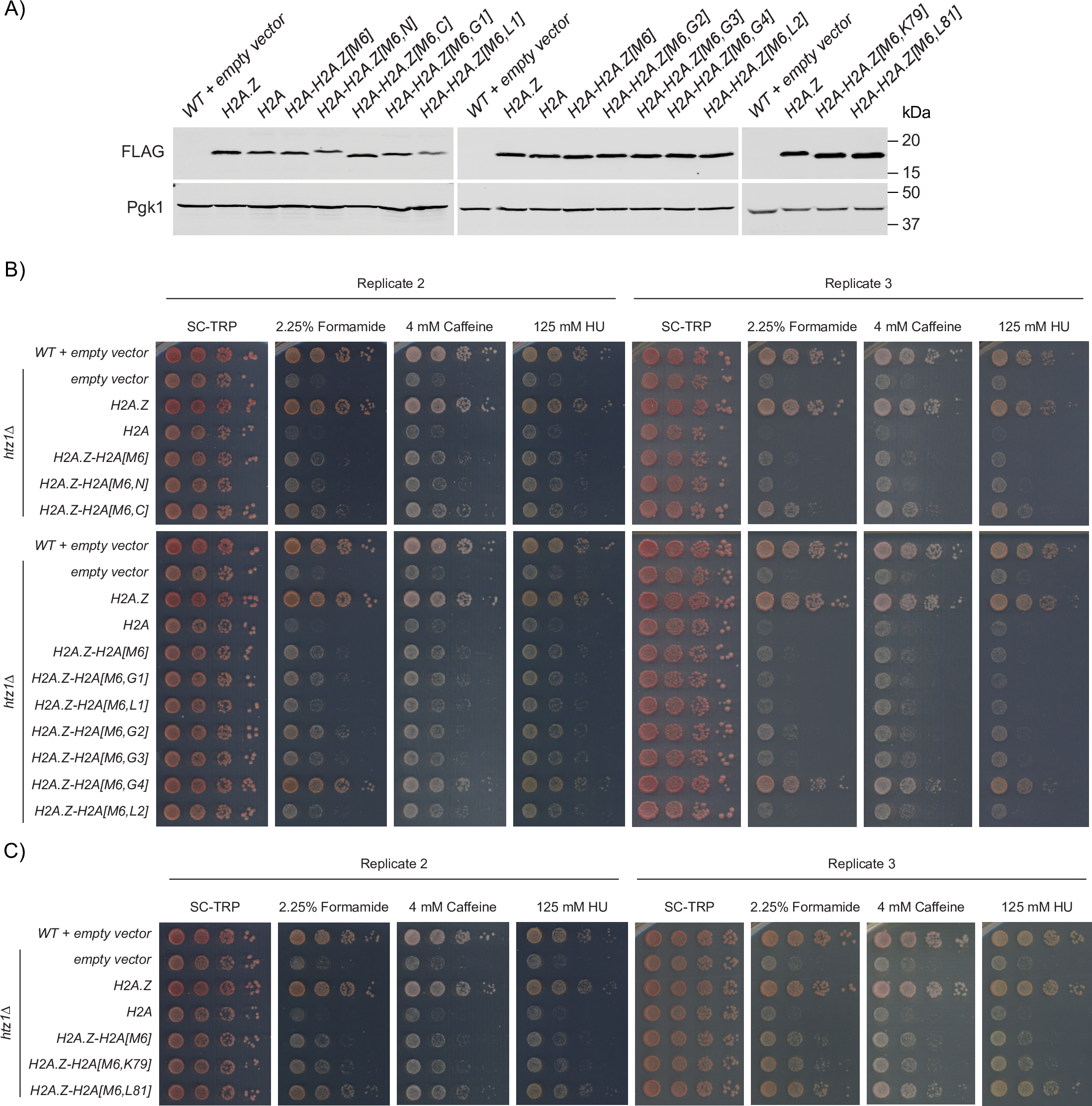

Supplement: S4 Fig — (A) Immunoblotting of whole-cell extracts indicated that all combination hybrid constructs were present in the mutants in similar levels as the H2A.Z construct. Pgk1 was used as a loading control. (B) Biological replicates of growth assays for Fig 4A. (C) Biological replicates of growth assays for Fig 4D. Cells expressing the indicated hybrid constructs were 10-fold serially diluted, spotted onto SC-TRP media with the indicated concentrations of formamide, caffeine, and hydroxyurea and grown for 3 days. (TIF) [file pgen.1009950.s004.tif]

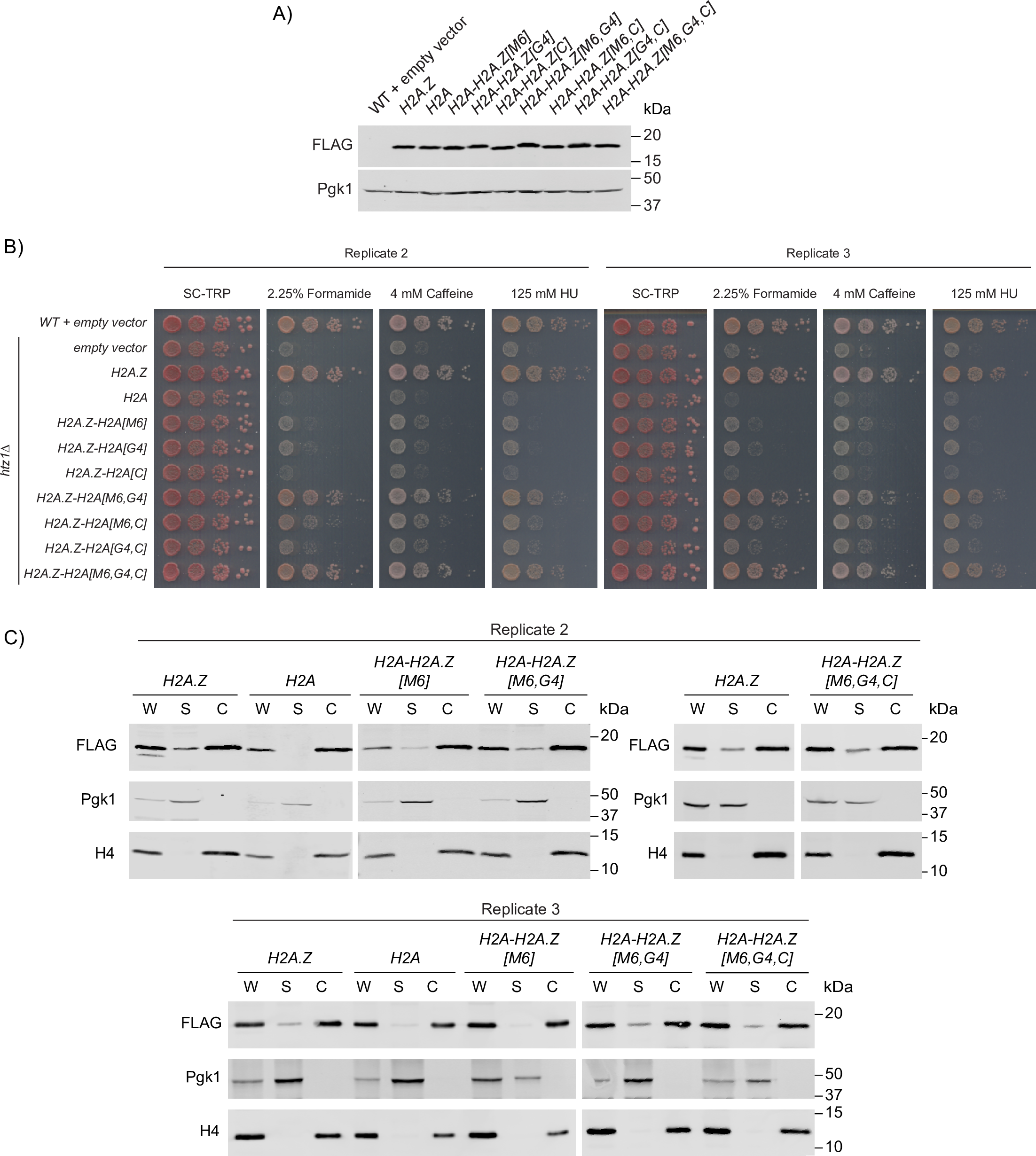

Supplement: S5 Fig — (A) Protein levels of C-terminal hybrid constructs from Fig 5A were analyzed by immunoblotting of whole-cell extracts with an anti-FLAG antibody, with Pgk1 was used as a loading control. (B) Biological replicates of growth assays for Fig 5A. Cells expressing the indicated hybrid constructs were 10-fold serially diluted, spotted onto SC-TRP media with the indicated concentrations of formamide, caffeine, and hydroxyurea and grown for 3 days. (C) Biological replicates of chromatin association assay in Fig 5B. Whole-cell extracts (W) were separated into chromatin (C) and soluble (S) (non-chromatin) fractions and analyzed by immunoblotting. FLAG antibodies detected the hybrid constructs, while H4 and Pgk1 were used as controls for the chromatin and soluble fractions, respectively. (TIF) [file pgen.1009950.s005.tif]

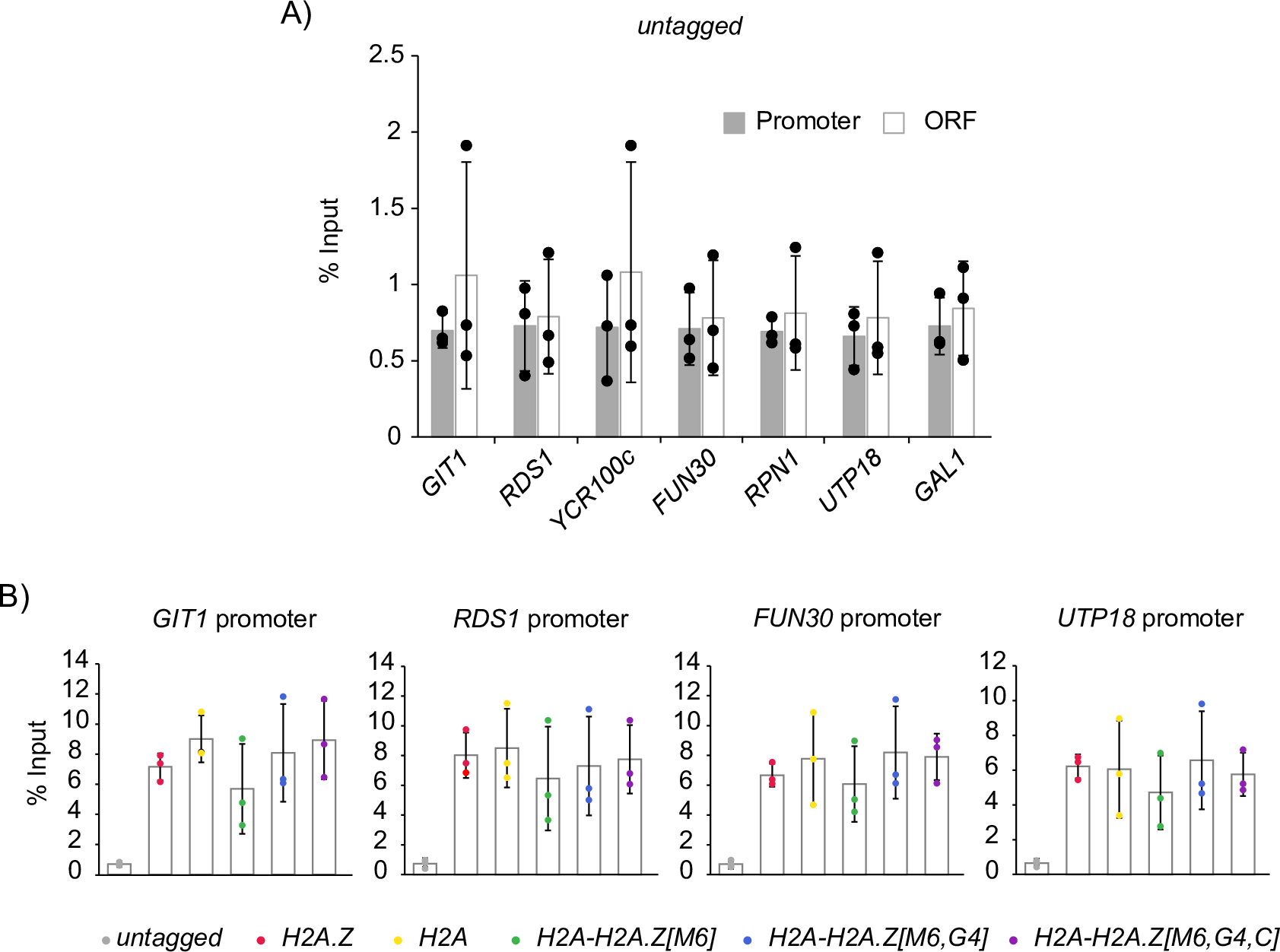

Supplement: S6 Fig — (A) FLAG-tagged hybrid enrichment levels for the untagged control determined by ChIP-qPCR for three replicates were normalized to their respective inputs. (B) Enrichment of all constructs at promoter loci that were relatively enriched for H2A.Z in comparison to the gene ORF (see Fig 6A). While all constructs were enriched over the untagged control (P > 0.05) all other comparisons were non-significant as determined by unpaired two-tailed Student’s t-tests. (TIF) [file pgen.1009950.s006.tif]

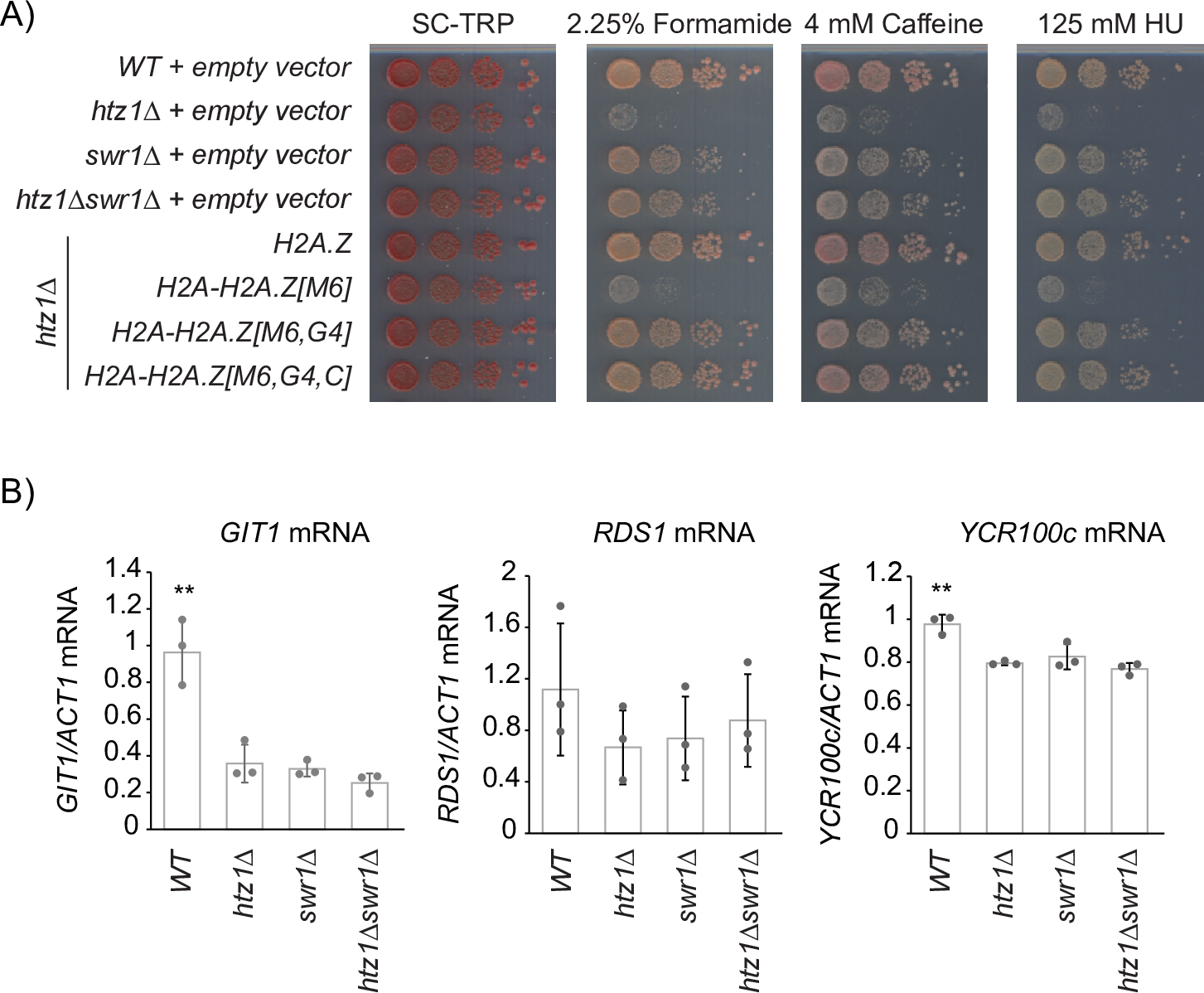

Supplement: S7 Fig — (A) The H2A-H2A.Z[M6,G4] and H2A-H2A.Z[M6,G4,C] mutants in a htz1Δ background had improved growth in comparison to the swr1Δ or swr1Δhtz1Δ mutants and had similar growth phenotypes to the H2A.Z mutant. Cells expressing the indicated hybrid constructs were 10-fold serially diluted, spotted onto SC-TRP media with the indicated concentrations of formamide, caffeine, and hydroxyurea and grown for 3 days. (C) Decrease in mRNA levels in the htz1Δ mutant for heterochromatin-proximal genes was not caused by the presence of apo-SWR1-C. RT-qPCR analysis of heterochromatin-proximal genes mRNA levels from three replicates were normalized to ACT1 mRNA levels. Error bars indicate the standard deviation between the three replicates. Significant comparisons determined by unpaired two-tailed Student’s t-tests are indicated: ** = p-value <0.01. (TIF) [file pgen.1009950.s007.tif]

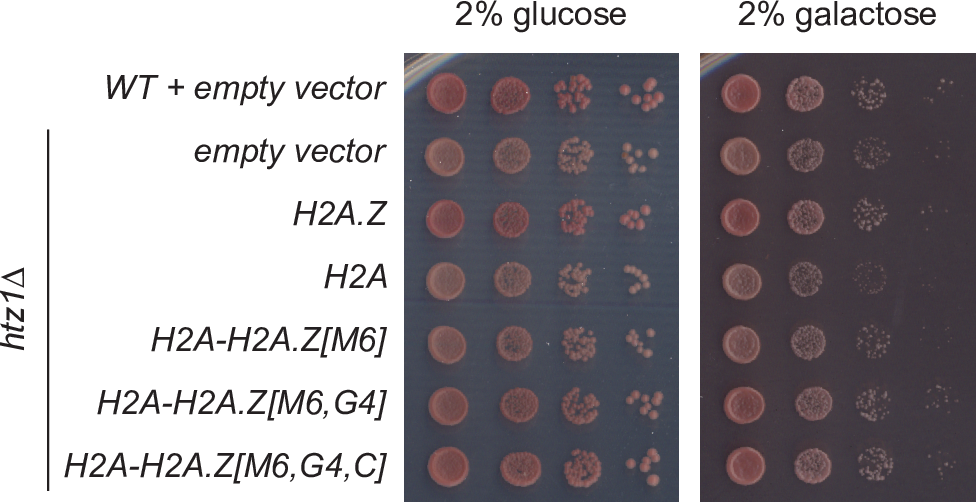

Supplement: S8 Fig — Cells expressing the indicated hybrid constructs were 10-fold serially diluted, spotted onto SC-TRP media containing either 2%-glucose or 2%-galactose with 0.02 mg/mL ethidium bromide and grown for 3 days. (TIF) [file pgen.1009950.s008.tif]
